# Supplementary material for: Single-cell multi-omics analysis identifies two distinct phenotypes of newly-onset microscopic polyangiitis
Source: Nat Commun. 2023 Oct 11;14:5789. doi: 10.1038/s41467-023-41328-0 (PMC10567716; doi:10.1038/s41467-023-41328-0)
Supplement: Supplementary file 1 — Supplementary Information [file 41467_2023_41328_MOESM1_ESM.pdf]

Supplementary information for:

Single-cell multi-omics analysis identifies two distinct phenotypes of newly-onset microscopic polyangiitis

Correspondence to:

Masayuki Nishide (nishide@imed3.med.Osaka-u.ac.jp), Department of Respiratory Medicine and Clinical Immunology, Graduate School of Medicine, Osaka University, Suita, Osaka, Japan.

Atsushi Kumanogoh (kumanogo@imed3.med.osaka-u.ac.jp), Department of Respiratory Medicine and Clinical Immunology, Graduate School of Medicine, Osaka University, Suita, Osaka, Japan.

This file includes supplementary tables 1 – 11 and supplementary figures 1-13.

Supplementary Table 1.
Characteristics of eight patients with microscopic polyangiitis and seven healthy donors recruited for CITE-seq and CyTOF.

Patients with MPA

	Patient No.		1	2	3	4	5	6	7	8
	Age		79	81	44	69	70	81	52	75
	Sex		Female	Male	Male	Female	Male	Male	Female	Female
	Medication		None	None	None	None	None	None	None	None
	New/Worse/Persistent		New	New	New	New	New	New	New	New
	Consideration when applying classification criteria		*Described in the Methods "Patient profiles" section							
Classification criteria for MPA	Nasal involvement		-3	-	-	-	-	-3	-	-
	MPO-ANCA positive		6	6	6	6	6	6	6	6
	Interstitial lung disease		3	3	-	3	3	3	3	-
	Pauci-immune glomerulonephritis on biopsy		-	-	3	-	-	-	-	3
	PR3-ANCA positive		-	-	-	-	-	-	-	-
	Blood eosinophil count		-	-	-	-	-	-	-	-
	SUM		6	9	9	9	9	6	9	9
BVAS	General		3	3	0	2	0	0	1	0
	Cutaneous		0	0	0	0	0	0	2	0
	Mucous membranes/Eyes		0	0	0	0	0	6	0	0
	ENT		6	0	0	6	0	6	0	3
	Chest		4	0	0	0	3	0	0	0
	Cardiovascular		0	0	0	0	0	0	0	0
	Renal		0	10	12	0	6	0	0	0
	Nervous system		0	0	0	0	9	0	6	9
	SUM		13	13	12	8	18	12	9	12
Blood Count	White Blood Cells	/μL	13180	10440	7210	6670	9810	9070	8320	13110
	Neutrophils	/μL	11190	9072	4333	5616	7308	6095	5366	11379
	Lymphocytes	/μL	1002	606	2278	680	1884	2222	1473	1468
	Monocytes	/μL	764	355	490	267	432	590	291	223
	Eosinophils	/μL	185	376	58	100	147	136	1173	13
	Basophils	/μL	40	31	50	7	39	27	17	26
	Hemoglobin	g/dL	9.4	7.5	12.2	6.5	12.0	12.6	10.7	9.6
	Pletelets	$\times 10^4$/μL	50.6	35.9	27	29.8	39.5	26.1	37.6	36.6
Urine	Occult Blood (Semi-Quantitative)	mg/dL	N.D.	0.20	0.50	0.03	0.20	0.06	N.D.	0.03
	Up/Ucr	mg/g Cr	0.13	0.37	0.98	N.D.	N.D.	0.16	0.14	0.09
Biochemical	Urea Nitrogen	mg/dL	10	14	37	15	18	21	7	12
	Creatinine	mg/dL	0.61	1.11	2.45	0.67	1.00	1.18	0.51	0.54
	eGFR	mL/min/1.73 m^2	70.3	49.4	24.7	65.9	57.3	46.0	96.4	81.5
	CRP	mg/dL	11.37	13.44	< 0.04	8.92	5.90	4.04	3.89	7.87
Immunological	MPO-ANCA	IU/mL	226.0	1730.0	1130.0	17.2	1690.0	22.0	261.0	76.5
	PR3-ANCA	IU/mL	2.2	< 1.0	< 1.0	< 1.0	1.4	< 1.0	< 1.0	< 1.0

Healthy Donors

Sample No.	1	2	3	4	5	6	7
Age	27	36	64	62	33	62	70
Sex	Male	Female	Female	Female	Male	Female	Male

MPA; Microscopic polyangiitis, BVAS; Birmingham Vasculitis Activity Score 2008 ver.3, ENT; Eyes, nose, and throat, Up/Ucr; Urine protein/urine creatinine ratio, eGFR; Estimated glomerular filtration rate, CRP; C-reactive protein, MPO; Myeloperoxidase, PR3; Proteinase 3, ANCA; Anti-neutrophil cytoplasmic antibody, N.D.; Not detected.

Supplementary Table 2.
Pseudo-bulk differential expressed genes (DEG) analysis in PBMCs derived from patients with MPA and healthy donors.

a

Gene Name	Fold Change	*P*-value
SOCS3	5.53	2.20E-03
S100A12	4.78	1.53E-02
S100A8	4.55	1.62E-02
S100A9	3.29	3.89E-03
MT-ATP8	3.04	3.79E-02
PTPRCAP	2.93	3.33E-02
PLBD1	2.88	3.22E-03
RPS10	2.56	4.41E-02
FPR1	2.43	9.87E-03
NAMPT	2.37	4.20E-02
VCAN	2.19	6.40E-03
IFI6	2.01	5.04E-02
BCL2A1	1.96	8.09E-02
GCA	1.87	7.75E-04
PIM3	1.86	5.13E-02
FCGR2A	1.86	1.28E-02
CD14	1.86	1.62E-02
GLUL	1.85	3.15E-03
CD69	1.83	6.63E-02
NEAT1	1.82	6.12E-02
SERPINA1	1.81	8.27E-03
SLC11A1	1.81	3.11E-03
IFITM3	1.80	4.93E-02
TNFSF13B	1.79	8.72E-03
BLVRB	1.78	2.57E-03
LYZ	1.78	1.54E-02
TSPO	1.77	1.35E-03
TIMP1	1.76	2.93E-04
LILRA5	1.76	2.02E-02
TALDO1	1.75	2.16E-04
PGD	1.73	2.18E-03
IFI30	1.72	4.48E-03
LILRB3	1.71	1.73E-02
LGALS1	1.70	1.99E-03
PLSCR1	1.70	1.52E-02
MNDA	1.69	2.77E-02
PIM1	1.67	2.01E-02
VAMP5	1.67	3.48E-02
NFKBIA	1.67	1.02E-01
GAPDH	1.66	1.09E-04
S100A11	1.66	9.36E-03
CTSD	1.65	4.60E-03
S100A6	1.65	2.21E-02
CAPG	1.64	4.28E-05
BCL3	1.64	7.43E-02
MSRB1	1.63	9.14E-03

Gene Name	Fold Change	*P*-value
MT2A	1.62	3.23E-02
SLC2A3	1.62	3.62E-02
PRDM1	1.62	4.24E-02
PYCARD	1.62	3.61E-03
RAB31	1.62	6.82E-03
FKBP5	1.62	6.23E-03
TESC	1.61	1.06E-03
FCN1	1.61	1.07E-02
UBALD2	1.60	7.48E-02
SERPINB1	1.60	3.34E-04
CEBPD	1.60	3.79E-02
EPSTI1	1.60	6.11E-02
SOD2	1.59	4.56E-02
IGSF6	1.59	4.23E-02
TIMP2	1.59	1.39E-02
GRN	1.59	2.50E-03
GPRIN3	1.58	2.81E-02
AGTRAP	1.58	2.74E-03
TYMP	1.58	1.19E-02
MS4A6A	1.57	2.28E-02
NAIP	1.57	1.93E-02
IFITM2	1.57	3.35E-03
UPP1	1.57	1.83E-03
CTSB	1.56	1.75E-03
C19orf38	1.56	6.44E-03
LGALS3	1.55	4.71E-03
GNG10	1.55	2.19E-03
ISG15	1.55	3.32E-02
VIM	1.55	1.98E-03
CD63	1.54	3.58E-03
BLVRA	1.54	3.94E-03
TNFSF10	1.54	1.74E-02
CEBPB	1.54	6.56E-02
S100A10	1.54	1.02E-02
SQOR	1.53	9.04E-04
CTSA	1.53	7.93E-04
H2AFJ	1.52	3.23E-03
NFKBIZ	1.52	5.37E-02
FOS	1.52	1.88E-01
MYC	1.51	1.09E-01
S100A4	1.51	1.47E-02
AIF1	1.51	3.84E-02
CD302	1.51	3.44E-02
NCF4	1.51	1.44E-03
C4orf48	1.50	2.55E-02
DDIT4	1.50	3.19E-02

b

CD14 Mono-Signature genes	Interferon-Signature genes
MS4A6A	ISG15
NAIP	IFITM3
LILRB3	IFITM2
RAB31	IFI6
PLSCR1	NFKBIA
CD14	CD69
SLC11A1	SOCS3
FPR1	SOD2
CD302	TNFSF10
AIF1	PLSCR1
LILRA5	PIM1
BLVRA	UPP1
CEBPD	IFI30
SOD2	VAMP5
S100A8	MT2A
LYZ	EPSTI1
FCN1	IFI44
MNDA	IFI44L
S100A9	
S100A12	
VCAN	
CTSD	
NEAT1	
IGFS6	
CAPG	
IFI30	
TYMP	
EPSTI1	
TNFSF10	
TSPO	
SERPINB1	
TALDO1	
FCGR2A	
S100A4	
PLBD1	
SLC2A3	
CTSA	
FOS	
NFKBIA	
PYCARD	

(a) The top 100 genes significantly upregulated in PBMCs derived from patients with MPA compared to healthy donors. *P*-values are calculated using two-sided Wilcoxon Rank Sum test. **(b)** A list of the genes related to $CD14^{+}$ monocyte signature genes and interferon signature genes.

Supplementary Table 3.
List of highly expressed genes in B cells derived from patients in MPA-IFN group compared to patients in MPA-MONO group.

Gene Name	Fold Change	*P*-value
MTRNR2L12	1.22	1.70E-87
MT-ATP6	1.07	4.78E-80
RPS10	0.858	5.28E-71
HLA-DPB1	0.848	1.41E-124
IGKC	0.729	5.83E-08
TCL1A	0.712	1.79E-35
HLA-DPA1	0.672	3.24E-81
IFI44L	0.653	6.72E-40
HLA-DQB1	0.593	7.61E-49
IFITM1	0.565	7.41E-28
IGHD	0.553	1.62E-29
LY6E	0.526	1.96E-33
TSPAN13	0.514	3.52E-41
HLA-DRA	0.504	1.35E-75
IGHM	0.491	2.47E-22
PCBP2	0.482	1.32E-21
HLA-DQA1	0.480	4.26E-37
XAF1	0.477	5.31E-24
CD37	0.430	4.69E-41
MX1	0.428	8.64E-22
RNASE6	0.425	1.68E-25
HLA-DMA	0.418	7.60E-28
HVCN1	0.417	6.11E-24
MT-ND4	0.409	7.97E-25
CD79B	0.397	2.28E-22
SELL	0.372	5.61E-16
IFITM2	0.350	2.95E-18
HLA-DOB	0.350	6.89E-19
NAPSA	0.343	1.36E-23
TRBC2	0.343	4.58E-14
FCMR	0.341	7.06E-16
CORO1A	0.339	4.45E-16
HLA-DMB	0.330	1.92E-16
POLD4	0.325	3.84E-13
MT-CO2	0.321	1.60E-12
HLA-DRB1	0.314	2.63E-25
SRSF5	0.309	1.24E-16
NIBAN3	0.308	4.73E-09
RPS4X	0.301	4.80E-14
CD72	0.297	7.94E-11
FCRL1	0.293	1.23E-11
SP110	0.291	4.20E-11
SNHG5	0.290	7.17E-11
BIRC3	0.284	1.40E-09
PTPN6	0.283	1.86E-10
CD22	0.281	4.96E-09

The top 50 genes significantly upregulated in B cells derived from patients in MPA-IFN group compared to patients in MPA-MONO group. *P*-values are calculated using two-sided Wilcoxon Rank Sum test.

Supplementary Table 4.
Clinical course of patients MPA-1, MPA-3, and MPA-5.

			MPA-MONO		MPA-IFN		MPA-IFN	
			Before-treatment	After-treatment	Before-treatment	After-treatment	Before-treatment	After-treatment
	Patient No.		1	1	3	3	5	5
	Age		79	79	44	45	70	70
	Sex		Female	Female	Male	Male	Male	Male
	Medication		None	GC + MMF	None	GC + IVCY -> MMF	None	GC + IVCY
	Duration of treatment (months)		0	4	0	12	0	2
	New/Worse/Persistent		New	Worse	New	Persistent	New	Persistent
BVAS	General		3	0	0	0	0	0
	Cutaneous		0	0	0	0	0	0
	Mucous membranes/Eyes		0	3	0	0	0	0
	ENT		6	3	0	0	0	0
	Chest		4	0	3	0	3	0
	Cardiovascular		0	6	0	0	0	0
	Renal		0	0	6	4	6	0
	Nervous system		0	0	9	0	9	3
	SUM		13	12	18	4	18	3
Blood Count	White Blood Cells	/μL	13180	15010	9810	9260	9810	8160
	Neutrophils	/μL	11190	13734	7308	8278	7308	7254
	Lymphocytes	/μL	1002	826	1884	426	1884	693
	Monocytes	/μL	764	375	432	491	432	204
	Eosinophils	/μL	185	45	147	19	147	0
	Basophils	/μL	40	30	39	46	39	8
	Hemoglobin	g/dL	9.4	13.9	12.0	10.8	12.0	12.8
	Pletelets	$\times 10^4$/μL	50.6	29.7	39.5	31.0	39.5	15.0
Urine	Occult Blood (Semi-Quantitative)	mg/dL	N.D.	N.D.	0.20	0.03	0.20	0.10
	Up/Ucr	mg/g Cr	0.13	0.07	N.D.	0.31	N.D.	N.D.
Biochemical	Urea Nitrogen	mg/dL	10	27	18	38	18	25
	Creatinine	mg/dL	0.61	0.84	1.00	2.03	1.00	0.92
	eGFR	mL/min/1.73 m^2	70.3	49.5	57.3	30.2	57.3	62.8
	CRP	mg/dL	11.37	4.55	5.90	0.04	5.90	< 0.04
Immunological	MPO-ANCA	IU/mL	226	5	1690	266	1690	264
	PR3-ANCA	IU/mL	2.2	< 1.0	1.4	N.A.	1.4	N.A.

MPA; Microscopic polyangiitis, GC; glucocorticoids, MMF; mycophenolate mofetil, IVCY; intravenous cyclophosphamide, BVAS; Birmingham Vasculitis Activity Score 2008 ver.3, ENT; Eyes, nose, and throat, Up/Ucr; Urine protein/urine creatinine ratio, eGFR; Estimated glomerular filtration rate, CRP; C-reactive protein, MPO; Myeloperoxidase, PR3; Proteinase 3, ANCA; Anti-neutrophil cytoplasmic antibody, N.A.; Not assessed, N.D.; Not detected.

Supplementary Table 5.
List of highly expressed genes in CD14 Mono_Activated and CD14 Mono_ISG subsets.

CD14 Mono_Activated

Gene Name	Fold change	Adjusted *P*-value
S100A8	1.29	*
S100A12	1.19	*
NAMPT	0.823	*
EGR1	0.781	2.49E-187
S100A9	0.740	*
BCL2A1	0.678	1.50E-278
FOS	0.674	1.22E-170
RGS2	0.635	8.81E-261
PLBD1	0.633	2.81E-224
ALOX5AP	0.600	1.46E-280
IER2	0.556	1.56E-121
DUSP1	0.527	2.33E-138
SRGN	0.508	6.80E-229
S100A6	0.506	*
SLC2A3	0.479	2.18E-174
CYP1B1	0.471	2.74E-180
SOD2	0.449	5.55E-101
KLF6	0.430	2.97E-110
MCL1	0.420	1.44E-98
H1FX	0.417	3.90E-32
BTG2	0.408	1.38E-64
H3F3B	0.406	5.24E-119
NCF1	0.406	5.06E-158
RBP7	0.398	2.10E-117
JUN	0.398	1.79E-70
PLAUR	0.395	1.37E-75
FPR1	0.391	7.26E-118
CTSD	0.387	8.25E-150
CDC42EP3	0.373	4.87E-103
ZFP36	0.370	1.30E-63
STK17B	0.358	6.97E-99
GCA	0.351	4.07E-97
FOSB	0.349	5.48E-68
SGK1	0.346	1.00E-59
CEBPD	0.344	1.52E-92
G0S2	0.343	5.22E-116
SLC25A37	0.337	1.52E-69
TSPO	0.332	8.65E-128
S100A4	0.327	1.58E-143
MYL6	0.325	2.40E-100
SLC11A1	0.320	1.08E-79
BASP1	0.315	1.52E-100
CLU	0.310	9.01E-66
STXBP2	0.310	2.50E-69
NFIL3	0.309	5.25E-106
ATP2B1-AS1	0.304	5.66E-55
SERPINB1	0.303	2.47E-78
NFKBIA	0.301	1.67E-34
CYBA	0.301	5.51E-96
VCAN	0.301	1.36E-110
PADI4	0.300	4.36E-104
CDA	0.299	4.77E-78
DYSF	0.294	2.26E-98
CXCL8	0.291	4.08E-115
SELL	0.288	7.20E-76
ELOB	0.287	5.91E-90
PGD	0.280	7.53E-77
MCEMP1	0.279	1.08E-92
JUNB	0.278	1.31E-37
PPP1R15A	0.277	1.08E-57
HP	0.275	4.19E-113
BLOC1S1	0.273	6.44E-58
ANPEP	0.272	1.19E-66
FCER1G	0.271	2.59E-66
AC020916.1	0.271	1.63E-53
PLEK	0.270	9.41E-48
MAFB	0.268	2.02E-11
S100A11	0.268	3.41E-101
ACSL1	0.263	2.61E-68
H3F3A	0.259	7.67E-97
TRIB1	0.256	1.58E-75
TUBA1A	0.253	3.42E-30

CD14 Mono_ISG

Gene Name	Fold change	Adjusted *P*-value
IFI6	1.17	*
LY6E	1.10	*
IFITM3	1.07	*
IFI44L	0.986	*
MX1	0.959	*
ISG15	0.900	*
FOLR3	0.755	2.02E-203
XAF1	0.705	*
IFI44	0.645	*
EPSTI1	0.605	*
RNF213	0.604	*
TYMP	0.596	*
MX2	0.589	*
SIGLEC1	0.537	*
IFIT3	0.515	*
OAS2	0.497	*
TNFSF13B	0.479	2.87E-266
OAS1	0.467	*
OAS3	0.464	*
IRF7	0.456	*
VCAN	0.448	1.19E-217
PLSCR1	0.446	2.23E-262
APOBEC3A	0.426	9.57E-253
STAT1	0.424	5.60E-237
EIF2AK2	0.424	*
HERC5	0.417	*
LAP3	0.398	5.43E-225
SCO2	0.396	3.54E-201
IFITM2	0.392	2.41E-139
IFIT2	0.390	*
STAT2	0.370	1.69E-194
TMEM176B	0.369	1.53E-116
CD14	0.363	8.60E-136
PARP14	0.358	1.74E-192
TNFSF10	0.349	2.69E-137
TMEM176A	0.348	4.58E-116
BLVRA	0.341	2.84E-156
IFIT1	0.339	*
TMEM123	0.337	1.57E-152
LGALS9	0.334	2.07E-149
TRIM22	0.334	1.94E-165
CLEC12A	0.328	2.74E-126
ADAR	0.325	1.73E-146
MNDA	0.322	3.62E-147
SAMD9L	0.321	1.56E-229
PCBP2	0.319	6.17E-95
FCN1	0.319	4.34E-154
UBE2L6	0.318	1.62E-137
MT2A	0.309	2.71E-52
BST2	0.309	2.66E-119
OASL	0.303	*
LGALS2	0.298	1.38E-59
CMPK2	0.298	*
GRN	0.296	4.47E-113
PARP9	0.292	1.40E-174
SERPINA1	0.287	1.10E-98
AC020656.1	0.285	1.74E-73
IFI16	0.284	5.65E-105
SP110	0.283	7.01E-110
IFI35	0.280	7.49E-132
CTSS	0.279	2.97E-164
MS4A6A	0.274	1.41E-98
SMCHD1	0.270	7.27E-83
APLP2	0.266	1.18E-92
CFD	0.262	3.54E-82
SNHG5	0.254	1.09E-75
SHISA5	0.252	5.78E-104

* Adjusted P-values were smaller than 2E-308

Highly expressed genes in CD14 Mono_Activated and CD14 Mono_ISG were determined by DEG analysis in comparison with the $CD14^{+}$ monocyte population. Genes that shows 0.25 < Fold change were listed. *P*-values are calculated using two-sided Wilcoxon Rank Sum test.

Supplementary Table 6.
List of genes with altered expression before and after treatment for each case.

	MPA-1 (MPA-MONO)			MPA-3 (MPA-IFN)			MPA-5 (MPA-IFN)		
	Gene Name	Fold Change	Adjusted *P*-value	Gene Name	Fold Change	Adjusted *P*-value	Gene Name	Fold Change	Adjusted *P*-value
Upregulated genes After treatment	IL1R2	2.67	*	CEBPD	1.83	*	MT-ATP8	1.40	3.13E-173
	FKBP5	1.44	*	RPS2	1.67	*	RPLP0	1.26	8.44E-239
	CD163	1.43	*	CYBA	1.58	*	RPS3A	1.24	5.40E-291
	AL137857.1	1.20	*	S100A9	1.47	*	RPS4X	1.21	1.49E-265
	TXNIP	1.17	4.19E-263	AL133415.1	1.27	7.73E-303	RPS23	1.21	1.77E-290
	IFITM2	1.18	4.21E-225	SMAP2	1.27	*	RPL32	1.21	7.41E-282
	AC020656.1	1.11	2.09E-279	JUN	1.21	5.57E-207	EEF1A1	1.18	6.49E-291
	DDIT4	0.989	*	AL137857.1	1.16	*	RPS13	1.17	5.67E-273
	CALM2	0.979	*	TSC22D3	1.14	*	TXNIP	1.17	7.69E-179
	HMGB2	0.829	3.79E-181	FKBP5	1.11	*	RPL5	1.14	3.51E-236

	MPA-1			MPA-3			MPA-5		
	Gene Name	Fold Change	Adjusted *P*-value	Gene Name	Fold Change	Adjusted *P*-value	Gene Name	Fold Change	Adjusted *P*-value
Downregulated genes After treatment	MTRNR2L12	-2.17	*	MTRNR2L12	-1.94	*	MTRNR2L12	-1.69	2.78E-237
	RPL41	-1.70	*	RPL41	-1.81	*	RPL41	-1.42	*
	H1FX	-1.21	1.74E-201	IFI44L	-0.773	5.94E-179	IFI6	-1.17	3.14E-154
	EGR1	-1.04	5.98E-153	PCBP2	-0.735	2.15E-187	SOCS3	-1.04	9.01E-150
	CLU	-1.00	8.24E-262	IFITM3	-0.700	9.93E-146	LY6E	-0.960	3.94E-127
	JUND	-0.903	8.17E-234	SIGLEC1	-0.475	1.42E-114	NAMPT	-0.858	9.16E-87
	PLAC8	-0.867	6.61E-163	CD300E	-0.399	3.72E-48	PIM3	-0.742	5.15E-99
	BCL2A1	-0.812	1.85E-185	IFITM1	-0.399	1.96E-50	ZFP36L1	-0.739	2.05E-52
	NCF1	-0.806	8.50E-164	MEF2C	-0.392	1.61E-42	VCAN	-0.701	1.02E-80
	SGK1	-0.787	2.67E-108	IFI6	-0.389	2.10E-60	ISG15	-0.652	2.22E-54

* Adjusted P-values were smaller than 2E-308

The top 10 upregulated and downregulated genes in $CD14^+$ monocytes, comparing pre- and post-treatment conditions in each case. *P*-values are calculated using two-sided Wilcoxon Rank Sum test.

Supplementary Table 7.
Characteristics of 43 patients with microscopic polyangiitis recruited for assessing clinical parameters.

	Patient No.		1	2	3	4	5	6	7	8	9	10	11	12	13	14	15	16	17	18	19	20	21	22
	Age		77	77	75	70	85	70	77	56	84	70	67	76	57	67	82	71	78	73	82	77	75	72
	Sex		Female	Female	Female	Male	Male	Male	Female	Female	Female	Male	Female	Male	Male	Male	Female	Female	Female	Male	Female	Female	Female	Male
	Medication		GC	None	GC/AZP	None	None	None	GC	None	GC	None	None	GC	None	GC	None	None	GC	None	GC/AZP	None	GC	GC/AZP
	New/Worse/Persistent		NEW	NEW	PER	NEW	NEW	NEW	NEW	NEW	PER	NEW	NEW	PER	NEW	NEW	NEW	NEW	PER	NEW	PER	NEW	WOR	WOR
Blood Count	White Blood Cells	/μL	11230	7660	8160	11500	4270	9810	12630	6570	13240	9110	5420	9630	11200	16790	14570	12620	10530	12420	6610	12900	11970	15320
	Neutrophils	/μL	8737	5033	6144	8660	2566	7308	9586	4586	11757	7060	3453	8773	8814	14557	12720	10942	7360	10097	5030	10010	10941	14018
	Lymphocytes	/μL	1853	1923	1053	1553	1349	1884	1806	1413	1112	1203	1350	510	1702	1461	918	934	2506	1466	1249	1767	634	444
	Monocytes	/μL	606	360	694	575	282	432	644	388	291	501	331	327	381	688	466	618	600	447	258	516	335	797
	Eosinophils	/μL	22	283	220	656	51	147	556	151	13	310	238	10	269	50	423	88	42	373	20	568	24	46
	Basophils	/μL	11	61	49	58	21	39	38	33	66	36	49	10	34	34	44	38	21	37	53	39	36	15
	Hemoglobin	g/dL	9.9	11.9	13.4	12.3	12.8	12	7.4	11.1	10.2	14.1	16.2	10.3	11.5	13.1	11.1	9.2	12.7	10.9	11.4	10.7	11.4	11.2
	Pletelets	× 10^4 /μL	199	441	302	351	192	395	608	370	333	347	233	144	283	284	370	419	206	322	267	580	451	320
Urine	Occult Blood (Semi-Quantitative)	mg/dL	1	N.D.	N.D.	1	0.1	0.2	N.A.	0.1	N.D.	0.5	N.A.	0.1	N.A.	0.03	0.2	0.1	N.A.	0.2	0.06	0.1	N.A.	1
	Up/Ucr	mg/g Cr	0	N.A.	0.56	0.52	N.A.	N.A.	N.A.	0.54	N.A.	0.24	N.A.	N.A.	N.A.	0.16	N.A.	N.A.	N.A.	1.24	N.A.	0.4	N.A.	N.A.
Biochemical	Urea Nitrogen	mg/dL	31	14	28	21	17	18	N.A.	15	20	14	13	45	12	16	11	16	21	34	15	11	16	28
	Creatinine	mg/dL	1.38	0.52	1.42	0.79	0.77	1	0.63	2.24	0.86	0.96	0.97	2.22	0.9	0.71	0.58	0.54	0.67	3.09	0.39	0.6	0.51	2.86
	eGFR	mL/min/1.73 m^2	29	84.3	28.3	74.2	72.2	57.3	68.3	18.7	47.4	59.9	44.3	23.4	68.2	84.4	73.4	82.8	63.6	16.5	113.4	72.1	86.7	18
	CRP	mg/dL	0.22	1.02	0.43	6.63	0.07	5.9	9.34	1.77	5.01	11.04	0.61	0.06	6.49	10.47	6.93	12.84	< 0.04	10.68	0.98	13.13	9.8	14.51
Immunological	MPO-ANCA	IU/mL	55.6	66.4	52.2	71	31.3	1690	144	N.A.	25.4	245	308	4	59.4	117	36.3	27.7	68.2	32.4	< 1.0	267	N.A.	11.5
	PR3-APCA	IU/mL	< 1.0	1.4	N.A.	< 1.0	< 1.0	1.4	< 1.0	< 1.0	2.1	2.7	< 1.0	N.A.	< 1.0	< 1.0	5.9	< 1.0	N.A.	< 1.0	< 1.0	< 1.0	181	< 1.0

	Patient No.		23	24	25	26	27	28	29	30	31	32	33	34	35	36	37	38	39	40	41	42	43
	Age		64	74	84	76	78	81	52	81	66	82	80	62	55	70	75	43	69	79	79	75	80
	Sex		Male	Male	Female	Male	Female	Female	Female	Female	Male	Female	Male	Male	Male	Female	Female	Male	Female	Female	Male	Female	Male
	Medication		GC	None	GC/AZP	GC	None	None	None	None	None	GC	None	None	GC	GC	GC/IVCY	None	None	None	None	None	None
	New/Worse/Persistent		WOR	NEW	WOR	WOR	NEW	NEW	NEW	NEW	NEW	WOR	NEW	NEW	WOR	NEW	PER	NEW	NEW	NEW	NEW	NEW	NEW
Blood Count	White Blood Cells	/μL	14160	9650	11990	9830	12840	17540	8320	10470	9840	11170	14040	14290	8660	9520	10670	7210	6670	17540	10440	13110	9070
	Neutrophils	/μL	13310	8357	10887	8434	11505	14102	5366	8376	8738	9226	12931	12361	7759	8644	9720	4333	5616	15348	9072	11379	6095
	Lymphocytes	/μL	609	820	588	649	1168	2543	1473	1215	846	1385	505	857	693	609	437	2278	680	1245	606	1468	2222
	Monocytes	/μL	227	357	492	678	167	456	291	440	246	559	590	915	199	209	437	490	267	807	355	223	590
	Eosinophils	/μL	0	97	12	39	0	386	1173	408	0	0	0	43	0	10	0	58	100	88	376	13	136
	Basophils	/μL	14	19	12	29	0	53	17	31	10	0	14	114	9	48	75	50	7	53	31	26	27
	Hemoglobin	g/dL	9.2	10.7	10.5	11.7	9.1	9.5	10.7	9.7	9.3	8.1	8.3	14.6	13.1	10.7	11.7	12.2	6.5	11.2	7.5	9.6	12.6
	Pletelets	× 10^4 /μL	376	564	325	203	553	388	376	343	364	444	368	544	365	336	266	270	298	596	359	366	261
Urine	Occult Blood (Semi-Quantitative)	mg/dL	0.1	0.2	N.A.	0.1	N.D.	0.1	N.A.	0.1	1	0.1	0.2	N.D.	N.D.	0.5	N.A.	0.5	0.03	N.A.	0.2	0.03	0.06
	Up/Ucr	mg/g Cr	N.A.	0.44	N.A.	0.13	N.A.	1.06	N.A.	1.23	N.A.	1	1.18	0.35	N.A.	N.A.	N.A.	0.98	N.A.	N.A.	0.37	0.09	0.16
Biochemical	Urea Nitrogen	mg/dL	22	23	N.A.	26	19	30	7	21	44	30	55	28	10	14	19	37	15	15	14	12	21
	Creatinine	mg/dL	0.91	1.39	0.61	1.06	0.72	2.53	0.51	1.36	0.98	1.44	2	0.85	0.78	0.56	0.54	2.45	0.67	0.66	1.11	0.54	1.18
	eGFR	mL/min/1.73 m^2	65.2	39.3	69	52.5	58.8	14.7	96.4	29	59.6	27.2	25.8	70.9	80.6	79.9	81.5	24.7	65.9	64.5	49.4	81.5	46
	CRP	mg/dL	1.64	6.32	4.54	N.A.	4.79	14.29	3.89	6.95	3.86	1.74	3.21	12.78	3.27	3.42	< 0.04	< 0.04	8.92	16.48	13.44	7.87	4.04
Immunological	MPO-ANCA	IU/mL	52.8	2900	< 1.0	162	270	311	261	60.8	832	140	201	22.7	7	5.4	< 1.0	1130	17.2	226	1730	76.5	22
	PR3-ANCA	IU/mL	< 1.0	N.A.	N.A.	N.A.	< 1.0	3.4	N.A.	< 1.0	< 1.0	< 1.0	< 1.0	< 1.0	N.A.	< 1.0	< 1.0	< 1.0	< 1.0	2.2	< 1.0	< 1.0	< 1.0

MPA; Microscopic polyangiitis, GC; Glucocorticoids, AZP;Azathioprine, IVCY; Intravenous cyclophosphamide, WOR; Worse, PER; Persistent, Up/Ucr; Urine protein/urine creatinine ratio, eGFR; Estimated glomerular filtration rate, CRP; C-reactive protein, MPO; Myeloperoxidase, PR3; Proteinase 3, ANCA; Anti-neutrophil cytoplasmic antibody. The table includes MPO-ANCA and PR3-ANCA levels if they were measured within one month prior to the date of blood collection, and there was no change in treatment during that period. N.A.; Not assessed, N.D.; Not detected.

Supplementary Table 8.
Correlation between the percentage of monocytes and representative clinical parameters, and between serum IFN-α concentrations and representative clinical parameters.

			v.s. Monocyte Ratio (% of PBMC)		v.s. Serum IFN-α (pg/mL)	
			R	*P*	R	*P*
Blood Count	White Blood Cells	/μL	0.30	0.0083	-0.11	0.36
	Neutrophils	/μL	0.34	0.0031	-0.16	0.17
	Lymphocytes	/μL	-0.50	0.000014	0.22	0.059
	Monocytes	/μL	0.41	0.00037	0.035	0.76
	Eosinophils	/μL	-0.13	0.27	0.21	0.063
	Basophils	/μL	-0.039	0.73	0.13	0.25
	Hemoglobin	g/dL	-0.054	0.64	-0.14	0.24
	Pletelets	$\times 10^4$/μL	0.042	0.71	0.23	0.049
Urine	Occult Blood (Semi-Quantitative)	mg/dL	0.19	0.15	0.092	0.49
	Up/Ucr	mg/g Cr	-0.073	0.60	0.36	0.011
Biochemical	Urea Nitrogen	mg/dL	0.17	0.16	-0.011	0.93
	Creatinine	mg/dL	0.033	0.77	-0.075	0.52
	eGFR	mL/min/1.73 m^2	-0.024	0.83	0.032	0.79
	CRP	mg/dL	0.30	0.0089	-0.052	0.67
Immunological	MPO-ANCA	IU/mL	-0.17	0.15	0.28	0.020
	PR3-ANCA	IU/mL	0.15	0.43	-0.087	0.68

Up/Ucr; Urine protein/urine creatinine ratio, eGFR; Estimated glomerular filtration rate, CRP; C-reactive protein, MPO; Myeloperoxidase, PR3; Proteinase 3, ANCA; Anti-neutrophil cytoplasmic antibody. Correlations and *P*-values were quantified using Kendall's correlation coefficient (R).

Supplementary Table 9.
List of Total-seq C antibodies for CITE-seq.

Target	Antibody	Catalog Number	Final conc. (ng/mL)
CD3	TotalSeq™-C0034 anti-human CD3 Antibody	300479	500
CD4	TotalSeq™-C0072 anti-human CD4 Antibody	300567	31
CD8a	TotalSeq™-C0080 anti-human CD8a Antibody	301071	16
CD11b	TotalSeq™-C0161 anti-human CD11b Antibody	301359	31
CD14	TotalSeq™-C0081 anti-human CD14 Antibody	301859	250
CD16	TotalSeq™-C0083 anti-human CD16 Antibody	302065	63
CD19	TotalSeq™-C0050 anti-human CD19 Antibody	302265	125
CD20	TotalSeq™-C0100 anti-human CD20 Antibody	302363	125
CD25	TotalSeq™-C0085 anti-human CD25 Antibody	302649	500
CD27	TotalSeq™-C0154 anti-human CD27 Antibody	302853	250
CD28	TotalSeq™-C0386 anti-human CD28 Antibody	302963	1500
CD38	TotalSeq™-C0389 anti-human CD38 Antibody	303543	4000
CD45	TotalSeq™-C0391 anti-human CD45 Antibody	304068	63
CD45RA	TotalSeq™-C0063 anti-human CD45RA Antibody	304163	16
CD45RO	TotalSeq™-C0087 anti-human CD45RO Antibody	304259	4000
CD123	TotalSeq™-C0064 anti-human CD123 Antibody	306045	500
HLA-DR	TotalSeq™-C0159 anti-human HLA-DR Antibody	307663	500
CD117	TotalSeq™-C0061 anti-human CD117 (c-kit) Antibody	313243	500
CD206	TotalSeq™-C0205 anti-human CD206 (MMR) Antibody	321147	1500
FcεRIα	TotalSeq™-C0352 anti-human FcεRIα Antibody	334645	1000
CD161	TotalSeq™-C0149 anti-human CD161 Antibody	339947	31
IgD	TotalSeq™-C0384 anti-human IgD Antibody	348245	188
CD294(CRTH2)	TotalSeq™-C0102 anti-human CD294 (CRTH2) Antibody	350131	500
CD127(IL-7Rα)	TotalSeq™-C0390 anti-human CD127 (IL-7Rα) Antibody	351356	250
CD197(CCR7)	TotalSeq™-C0148 anti-human CD197 (CCR7) Antibody	353251	250
CD196(CCR6)	TotalSeq™-C0143 anti-human CD196 (CCR6) Antibody	353440	500
CD183(CXCR3)	TotalSeq™-C0140 anti-human CD183 (CXCR3) Antibody	353747	1000
CD185(CXCR5)	TotalSeq™-C0144 anti-human CD185 (CXCR5) Antibody	356939	500
CD194(CCR4)	TotalSeq™-C0071 anti-human CD194 (CCR4) Antibody	359425	500
CD56(NCAM)	TotalSeq™-C0047 anti-human CD56 (NCAM) Antibody	362559	1000
CD33	TotalSeq™-C0052 anti-human CD33 Antibody	366633	16
CD11c	TotalSeq™-C0053 anti-human CD11c Antibody	371521	16
CD66b	TotalSeq™-C0166 anti-human CD66b Antibody	392909	250
CD45R(B220)	TotalSeq™-C0103 anti-mouse/human CD45R/B220 Antibody	103273	1000
CD138	TotalSeq™-C0055 anti-human CD138 (Syndecan-1) Antibody	356539	1000
CD5	TotalSeq™-C0138 anti-human CD5 Antibody	300637	250
CD10	TotalSeq™-C0062 anti-human CD10 Antibody	312233	500
CD24	TotalSeq™-C0180 anti-human CD24 Antibody	311143	125
CD80	TotalSeq™-C0005 anti-human CD80 Antibody	305243	1000
IgM	TotalSeq™-C0136 anti-human IgM Antibody	314547	125
IgG	TotalSeq™-C0375 anti-human IgG Fc Antibody	410727	250
CD21	TotalSeq™-C0181 anti-human CD21 Antibody	354923	63
PD-1	TotalSeq™-C0088 anti-human CD279 (PD-1) Antibody	329963	250

Supplementary Table 10.
Sequence information of each study participant.

Sequence ID	Study participant	Treatment	Estimated Number of Cells	Mean Reads per Cell	Median Genes per Cell	Number of Reads	Valid Barcodes	Sequencing Saturation	Q30 Bases in Barcode	Q30 Bases in RNA Read	Q30 Bases in UMI	Reads Mapped to Genome
1	MPA-1	Before treatment	7,635	46,164	1,563	352,459,721	92.10%	73.90%	95.30%	87.60%	94.50%	88.00%
2		After treatment	4,466	69,880	1,696	312,083,501	93.10%	84.80%	94.50%	88.30%	93.70%	88.30%
3	MPA-2	Before treatment	4,167	75,412	1,094	314,243,177	90.60%	81.30%	96.60%	90.70%	96.40%	86.00%
4	MPA-3	Before treatment	6,588	46,018	1,415	303,170,946	92.20%	83.10%	95.10%	93.50%	94.50%	94.20%
5		After treatment	6,893	57,207	1,678	394,327,325	93.20%	82.40%	94.40%	88.60%	92.70%	93.50%
6	MPA-4	Before treatment	8,076	79,116	1,744	638,940,819	91.00%	81.70%	96.00%	89.00%	95.60%	85.60%
7	MPA-5	Before treatment	6,872	53,547	1,570	367,975,449	91.50%	78.40%	94.50%	88.40%	93.70%	87.50%
8		After treatment	5,811	55,344	1,870	321,605,303	91.90%	77.90%	94.60%	88.60%	93.80%	92.50%
9	MPA-6	Before treatment	15,960	20,568	1,272	328,266,244	92.30%	55.50%	95.60%	87.00%	94.60%	86.70%
10	MPA-7	Before treatment	8,029	32,427	1,611	260,355,275	90.00%	72.00%	92.30%	91.10%	91.90%	94.60%
11	MPA-8	Before treatment	10,502	22,590	1,657	237,241,645	90.90%	63.60%	92.10%	90.20%	91.60%	93.30%
12	HD-1	Non	4,009	74,239	956	297,622,289	87.30%	63.80%	92.70%	90.60%	92.00%	92.60%
13	HD-2	Non	9,958	28,992	1,647	288,701,833	88.10%	63.90%	92.90%	91.00%	92.30%	93.60%
14	HD-3	Non	5,589	59,355	1,095	331,732,486	90.40%	75.80%	94.80%	90.80%	94.20%	91.40%
15	HD-4	Non	9,007	34,896	1,391	314,309,187	92.40%	79.80%	94.80%	90.50%	94.20%	89.00%
16	HD-5	Non	6,276	57,835	1,916	362,973,245	90.30%	80.60%	94.90%	91.10%	94.30%	92.40%
17	HD-6	Non	10,761	34,569	1,785	371,999,724	90.70%	69.80%	94.60%	90.20%	94.10%	89.40%
18	HD-7	Non	4,362	69,244	1,399	302,040,906	91.70%	81.40%	94.70%	88.80%	94.10%	83.50%

Sequence ID	Reads Mapped Confidently to Genome	Reads Mapped Confidently to Intergenic Regions	Reads Mapped Confidently to Intronic Regions	Reads Mapped Confidently to Exonic Regions	Reads Mapped Confidently to Transcriptome	Reads Mapped Antisense to Gene	Fraction Reads in Cells	Total Genes Detected	Median UMI Counts per Cell	Antibody: Number of Reads	Antibody: Mean Reads per Cell	Antibody: Valid Barcodes
1	65.80%	3.20%	9.80%	52.80%	48.00%	3.40%	87.60%	22,656	4,074	751,441,298	98,421	98.10%
2	76.20%	3.40%	8.20%	64.60%	60.50%	2.70%	84.10%	21,537	4,654	581,408,890	130,186	97.20%
3	53.00%	2.40%	4.30%	46.30%	42.50%	2.70%	90.80%	20,796	4,888	591,597,028	141,971	97.60%
4	83.90%	5.10%	11.20%	67.60%	62.90%	2.90%	82.00%	21,283	3,606	790,151,007	119,937	98.10%
5	75.50%	3.20%	8.10%	64.20%	60.30%	2.60%	89.20%	22,074	4,807	909,102,101	131,888	97.80%
6	64.00%	3.40%	9.80%	50.80%	46.40%	3.00%	81.40%	23,859	4,662	932,748,274	115,496	98.10%
7	66.60%	4.10%	11.00%	51.60%	47.80%	2.30%	83.00%	22,462	3,914	673,807,973	98,051	97.40%
8	69.20%	3.60%	7.20%	58.40%	54.20%	2.70%	87.70%	21,939	5,007	638,692,510	109,911	97.60%
9	67.10%	3.90%	13.70%	49.60%	44.80%	3.30%	89.00%	23,368	2,860	560,026,115	35,089	98.20%
10	76.50%	4.60%	8.30%	63.70%	57.50%	4.50%	94.30%	22,768	4,329	672,938,811	83,814	97.90%
11	80.00%	4.30%	8.10%	67.60%	61.20%	4.60%	94.60%	23,315	3,956	723,982,392	68,938	97.90%
12	59.30%	7.20%	12.80%	39.30%	31.20%	6.80%	32.20%	20,760	1,688	503,881,842	125,688	97.90%
13	73.80%	4.90%	10.20%	58.70%	51.20%	5.80%	88.80%	23,434	4,152	707,978,067	71,096	97.90%
14	75.60%	5.30%	10.30%	60.10%	53.40%	5.00%	44.30%	21,338	2,243	577,647,526	103,354	97.80%
15	77.50%	3.80%	8.70%	65.00%	59.60%	3.70%	93.20%	22,693	3,276	604,413,016	67,105	97.80%
16	76.90%	4.10%	10.80%	62.00%	56.40%	3.90%	92.90%	23,036	5,205	522,133,217	83,195	98.00%
17	74.10%	4.10%	10.60%	59.40%	53.90%	3.90%	93.00%	23,482	4,505	660,489,281	61,378	98.00%
18	66.80%	5.20%	15.10%	46.60%	42.90%	2.20%	81.00%	21,830	3,294	496,141,355	113,742	97.80%

Sequence ID	Antibody: Sequencing Saturation	Antibody: Q30 Bases in Barcode	Antibody: Q30 Bases in Antibody Read	Antibody: Q30 Bases in UMI	Antibody: Fraction Antibody Reads	Antibody: Fraction Antibody Reads Usable	Antibody: Antibody Reads Usable per Cell	Antibody: Fraction Antibody Reads in Aggregate Barcodes	Antibody: Fraction Unrecognized Antibody	Antibody: Antibody Reads in Cells	Antibody: Median UMIs per Cell
1	84.30%	95.60%	95.40%	94.40%	95.80%	31.90%	31,407	1.70%	4.20%	34.00%	4,223
2	85.70%	97.70%	0.00%	97.50%	94.00%	15.90%	20,715	3.40%	6.00%	17.30%	2,625
3	87.60%	97.20%	96.80%	96.50%	96.00%	17.10%	24,209	3.00%	4.00%	18.20%	2,138
4	88.00%	96.10%	79.50%	95.20%	96.10%	18.90%	22,694	6.90%	3.90%	20.40%	2,074
5	97.60%	0.00%	83.50%	97.40%	95.60%	19.20%	25,342	31.50%	4.40%	20.50%	3,127
6	73.30%	97.10%	96.30%	96.20%	95.70%	23.60%	27,213	3.70%	4.30%	25.30%	4,956
7	90.60%	97.80%	83.20%	97.50%	92.90%	29.70%	29,119	2.90%	7.10%	32.70%	2,291
8	91.70%	97.80%	82.30%	97.30%	92.70%	24.60%	26,989	4.00%	7.30%	27.10%	2,048
9	74.40%	95.70%	95.50%	94.70%	95.90%	45.90%	16,093	3.00%	4.10%	48.90%	3,310
10	77.30%	95.60%	73.80%	94.50%	95.80%	20.60%	17,255	7.80%	4.20%	22.10%	3,144
11	83.70%	95.60%	74.10%	94.60%	95.20%	35.30%	24,361	2.50%	4.80%	38.00%	3,242
12	88.90%	95.30%	79.20%	94.00%	95.50%	13.40%	16,890	1.30%	4.50%	14.40%	1,536
13	84.40%	95.40%	79.60%	94.00%	95.60%	28.70%	20,399	1.40%	4.40%	30.70%	2,350
14	61.20%	95.70%	67.00%	94.70%	95.30%	8.80%	9,069	1.80%	4.70%	9.40%	3,212
15	52.40%	95.80%	68.30%	94.80%	95.40%	13.90%	9,344	1.20%	4.60%	15.00%	4,080
16	46.40%	96.00%	69.30%	95.00%	95.70%	11.10%	9,221	0.30%	4.30%	11.80%	4,506
17	58.80%	95.90%	68.50%	94.80%	95.60%	19.30%	11,823	1.40%	4.40%	20.60%	4,419
18	46.90%	95.90%	69.50%	94.80%	95.60%	8.30%	9,389	2.40%	4.40%	8.90%	4,172

Supplementary Table 11.
List of heavy metal-conjugated antibodies for CyTOF.

PBMC mapping

Target	Clone	Label	Kit
CD19	HIB19	142Nd	Direct Immune Profiling Assay
CD4	RPA-T4	145Nd	Direct Immune Profiling Assay
CD8a	RPA-T8	146Nd	Direct Immune Profiling Assay
CD11c	Bu15	147Sm	Direct Immune Profiling Assay
CD16	3G8	148Nd	Direct Immune Profiling Assay
CD25	2A3	149Sm	Direct Immune Profiling Assay
MP1β	D21-1351	150Nd	Direct Immune Profiling Assay
CD123	6H6	151Eu	Direct Immune Profiling Assay
CD45	HI30	154Sm	Direct Immune Profiling Assay
CD14	M5E2	160Gd	Direct Immune Profiling Assay
CD69	FN50	162Dy	Direct Immune Profiling Assay
Foxp3	259D/C7	163Dy	Direct Immune Profiling Assay
CD161	HP3G10	164Dy	Direct Immune Profiling Assay
CD45RO	UCHL1	165Ho	Direct Immune Profiling Assay
CD27	O323	167Er	Direct Immune Profiling Assay
CD45RA	HI100	169Tm	Direct Immune Profiling Assay
CD3	UCHT1	170Er	Direct Immune Profiling Assay
CD57	HCD57	172Yb	Direct Immune Profiling Assay
CD127	A019D5	176Yb	Direct Immune Profiling Assay

Monocyte mapping

Target	Clone	Label	Kit
CD196/CCR6	9C4	141Pr	Direct Immune Profiling Assay
CD123/IL-3R	6H6	143Nd	Direct Immune Profiling Assay
CD19	HIB19	144Nd	Direct Immune Profiling Assay
CD4	RPA-T4	145Nd	Direct Immune Profiling Assay
CD8a	RPA-T8	146Nd	Direct Immune Profiling Assay
CD11c	Bu15	147Sm	Direct Immune Profiling Assay
CD16	3G8	148Nd	Direct Immune Profiling Assay
CD45RO	UCHL1	149Sm	Direct Immune Profiling Assay
CD45RA	HI100	150Nd	Direct Immune Profiling Assay
CD161	HP-3G10	151Eu	Direct Immune Profiling Assay
CD194/CCR4	L291H4	152Sm	Direct Immune Profiling Assay
CD25	BC96	153Eu	Direct Immune Profiling Assay
CD27	O323	154Sm	Direct Immune Profiling Assay
CD57	HCD57	155Gd	Direct Immune Profiling Assay
CD183/CXCR3	G025H7	156Gd	Direct Immune Profiling Assay
CD185/CXCR5	J252D4	158Gd	Direct Immune Profiling Assay
CD206	15-2	159Tb	Direct Immune Profiling Assay
CD28	CD28.2	160Gd	Direct Immune Profiling Assay
CD38	HB-7	161Dy	Direct Immune Profiling Assay
CD117	104D2	162Dy	Direct Immune Profiling Assay
CD56/NCAM	NCAM16.2	163Dy	Direct Immune Profiling Assay
TCRgd	B1	164Dy	Direct Immune Profiling Assay
FCεRIα	AER-37	165Ho	Direct Immune Profiling Assay
CD294	BM16	166Er	Direct Immune Profiling Assay
CD197/CCR7	G043H7	167Er	Direct Immune Profiling Assay
CD14	63D3	168Er	Direct Immune Profiling Assay
CD33	WM53	169Tm	Direct Immune Profiling Assay
CD3	UCHT1	170Er	Direct Immune Profiling Assay
CD20	2H7	171Yb	Direct Immune Profiling Assay
CD66b	G10F5	172Yb	Direct Immune Profiling Assay
HLA-DR	LN3	173Yb	Direct Immune Profiling Assay
IgD	IA6-2	174Yb	Direct Immune Profiling Assay
CD127	A019D5	176Yb	Direct Immune Profiling Assay
CD11b	ICRF44	209Bi	Direct Immune Profiling Assay

CD8 T cell mapping

Target	Clone	Label	Kit
CD19	HIB19	142Nd	Human PB Phenotyping panel
IL-5	TRFK5	143Nd	Human Intracellular Cytokine I Panel
IL-4	MP4-25D2	144Nd	Human Intracellular Cytokine I Panel
CD4	RPA-T4	145Nd	Human T-Cell Phenotyping panel
CD8a	RPA-T8	146Nd	Human T-Cell Phenotyping panel
CD11c	Bu15	147Sm	Human PB Phenotyping panel
CD16	3G8	148Nd	Human T-Cell Phenotyping panel
CD25	2A3	149Sm	Human T-Cell Phenotyping panel
MP1β	D21-1351	150Nd	Human Intracellular Cytokine I Panel
CD123	6H6	151Eu	Human PB Phenotyping panel
TNFα	Mab11	152Sm	Human Intracellular Cytokine I Panel
CD45	HI30	154Sm	Human T-Cell Phenotyping panel
IFN-α2b	7N4-1	155Gd	Human Intracellular Cytokine I Panel
IL-6	MQ2-13A5	156Gd	Human Intracellular Cytokine I Panel
IL-2	MQ1-17H12	158Gd	Human Intracellular Cytokine I Panel
GM-CSF	BVD221C11	159Tb	Human Intracellular Cytokine I Panel
CD14	M5E2	160Gd	Human PB Phenotyping panel
IL-17A	BL168	161Dy	Human Intracellular Cytokine I Panel
CD69	FN50	162Dy	Human T-Cell Phenotyping panel
Foxp3	259D/C7	163Dy	Human T-Cell Phenotyping panel
CD161	HP3G10	164Dy	Human PB Phenotyping panel
CD45RO	UCHL1	165Ho	Human T-Cell Phenotyping panel
IL-10	JES39D7	166Er	Human Intracellular Cytokine I Panel
CD27	O323	167Er	Human PB Phenotyping panel
IFN-γ	B27	168Er	Human Intracellular Cytokine I Panel
CD45RA	HI100	169Tm	Human T-Cell Phenotyping panel
CD3	UCHT1	170Er	Human T-Cell Phenotyping panel
Granzyme B	GB11	171Yb	Human Intracellular Cytokine I Panel
CD57	HCD57	172Yb	Human T-Cell Phenotyping panel
HLA-DR	L243	174Yb	Human T-Cell Phenotyping panel
Perforin	B-D48	175Lu	Human Intracellular Cytokine I Panel
CD127	A019D5	176Yb	Human T-Cell Phenotyping panel

Supplementary Fig. 1. UMAP plots of PBMCs derived from each study participant.

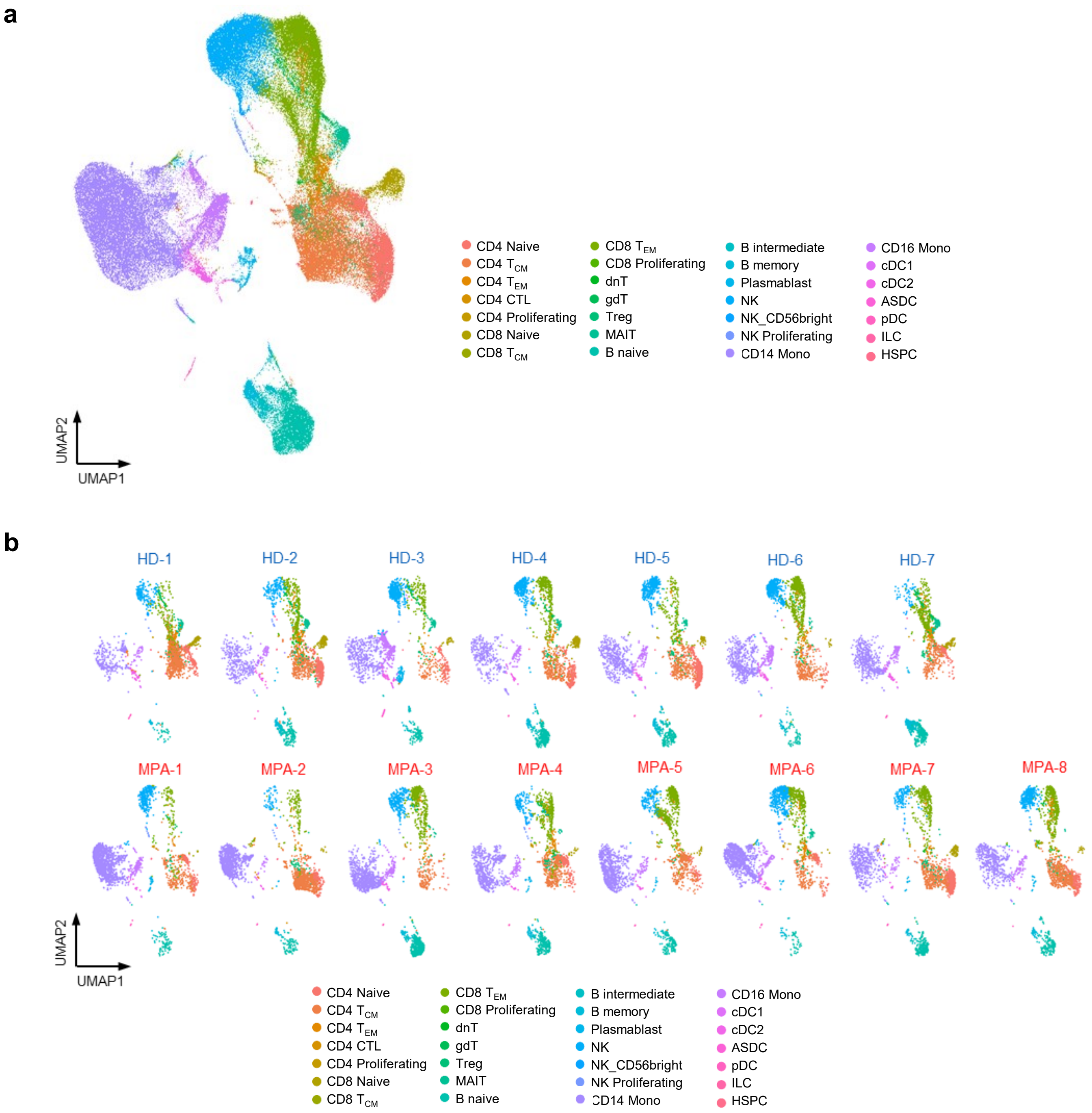

(a) UMAP plots showing CITE-seq data of 109,350 PBMCs derived from 15 total participants including healthy donors (n = 7) and patients with MPA (n = 8). Each cellular cluster was annotated with reference mapping. **(b)** Individual UMAP plots of PBMCs derived from each study participant.

Supplementary Fig. 2. CyTOF analysis of PBMCs derived from each study participant.

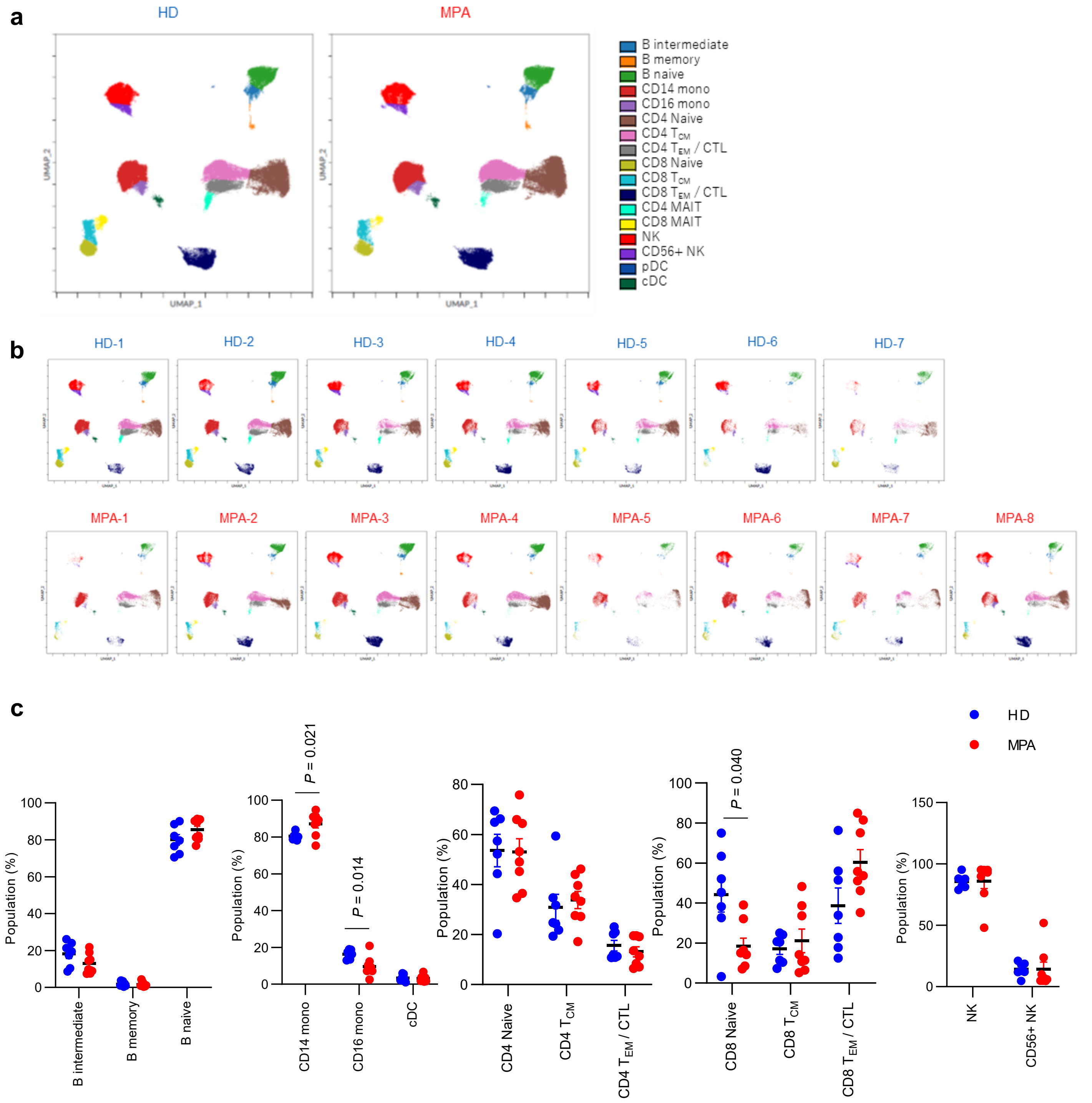

(a) UMAP plots showing CITE-seq data of 737,794 PBMCs derived from healthy donors (n = 7) and patients with MPA (n = 8). Each cellular cluster was manually annotated based on surface marker expression. The color-coded columns indicate cell types. **(b)** Individual UMAP plots of PBMCs derived from each study participant. Each cellular cluster was manually annotated based on surface marker expression. **(c)** Percentage of each cellular subpopulation relative to the total number of PBMCs. Blue dots indicate healthy donors (n = 7) and red dots indicate patients with MPA (n = 8). Values are means with SEMs and nominal *P*-values are calculated using a two-sided Mann-Whitney U test.

Supplementary Fig. 3. UMAP plots of monocytes derived from each study participant.

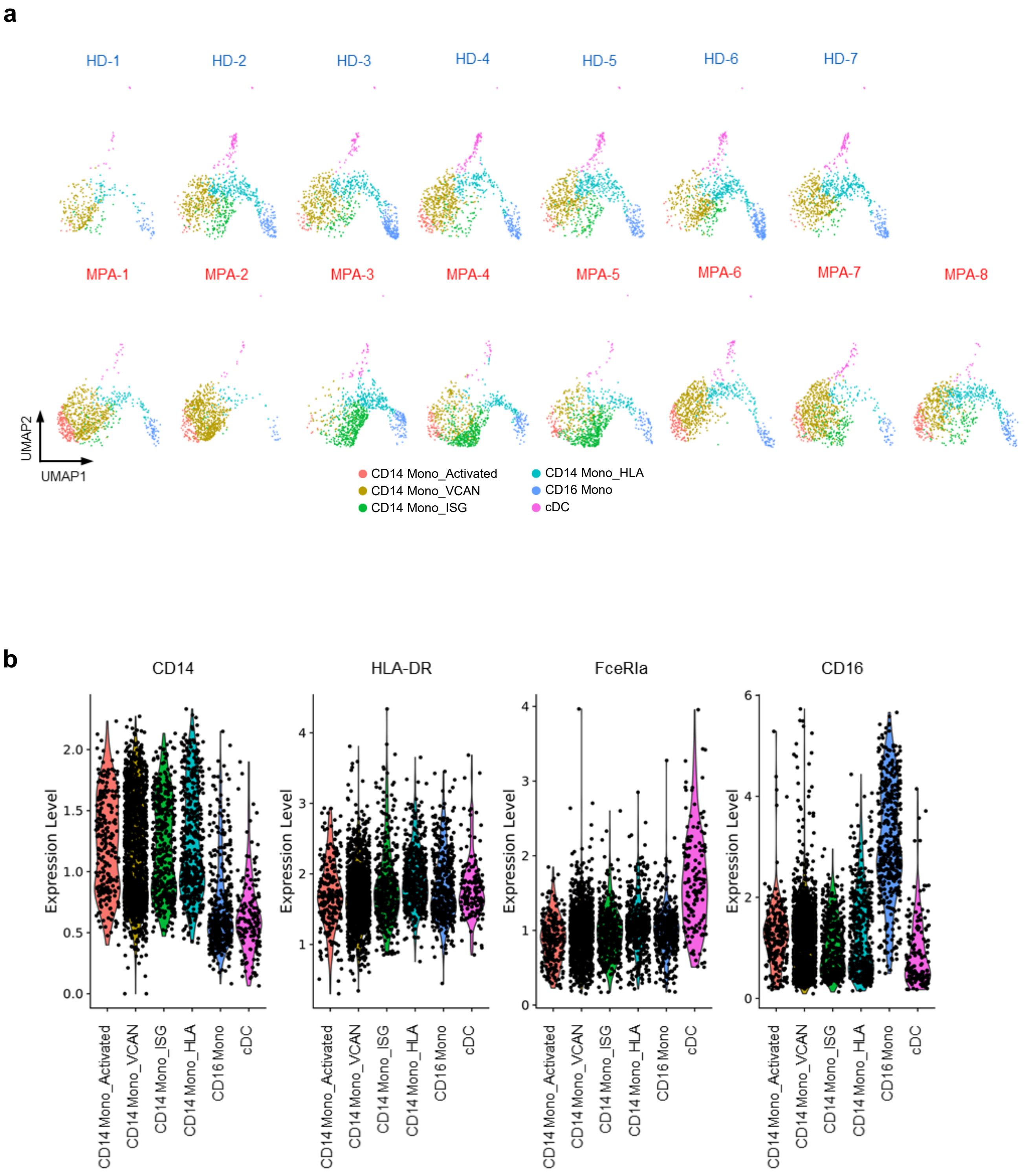

(a) UMAP plots showing the monocyte subpopulations in each study participant. Six cellular clusters were identified as shown in **Fig. 2a**. **(b)** Violin plots showing the scaled expression of representative cellular indexing of transcriptomes and epitopes (CITE) proteins that characterize each cellular cluster.

Supplementary Fig. 4. UMAP plots of CD8+ T cells derived from each study participant.

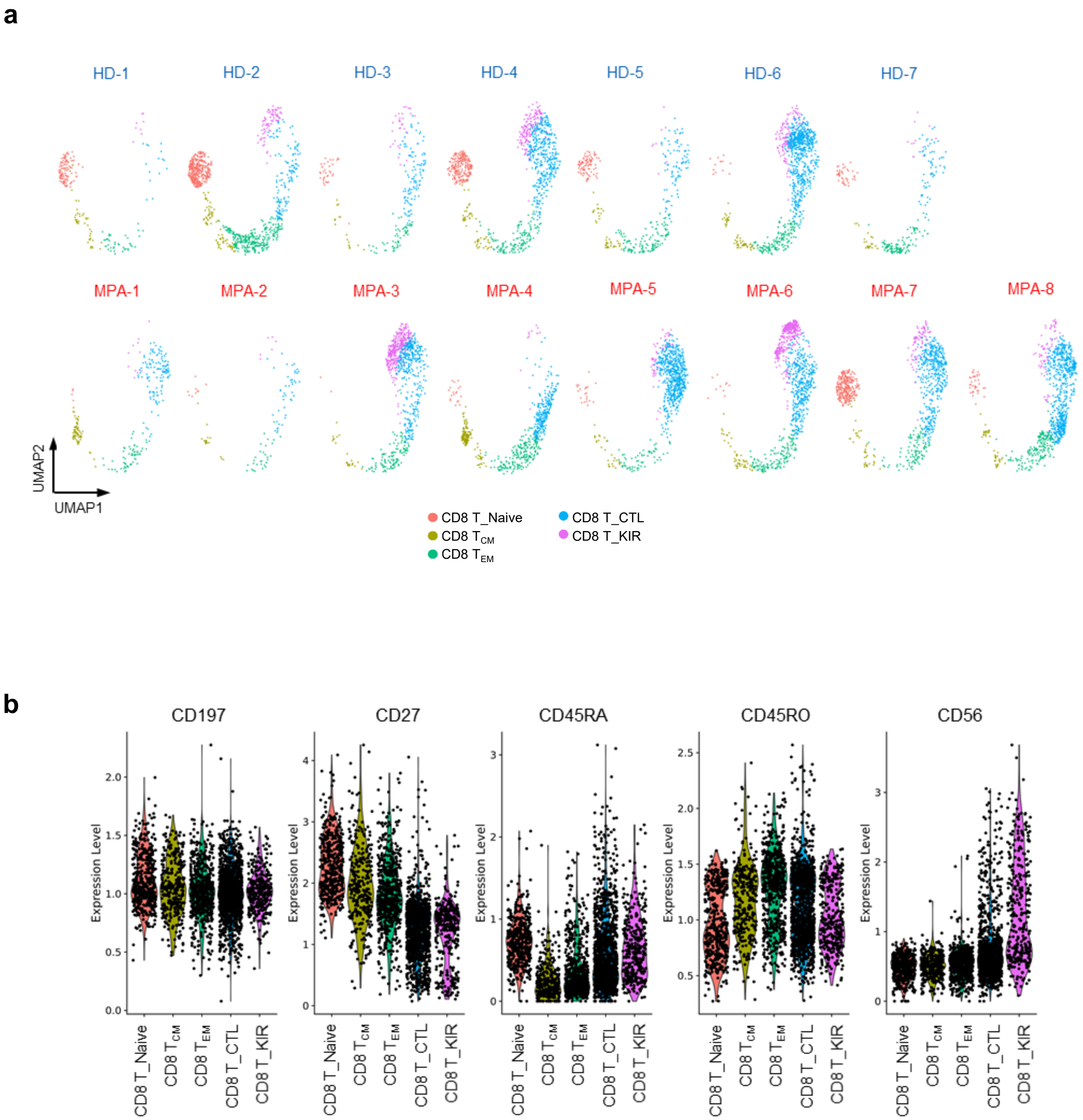

(a) UMAP plots showing the CD8$^+$ T cell subpopulations in each study participant. Five cellular clusters were identified as shown in **Fig. 2e**. **(b)** Violin plots showing the scaled expression of representative cellular indexing of transcriptomes and epitopes (CITE) proteins that characterize each cellular cluster.

Supplementary Fig.5. UMAP plots and differential abundance analysis of B cells.

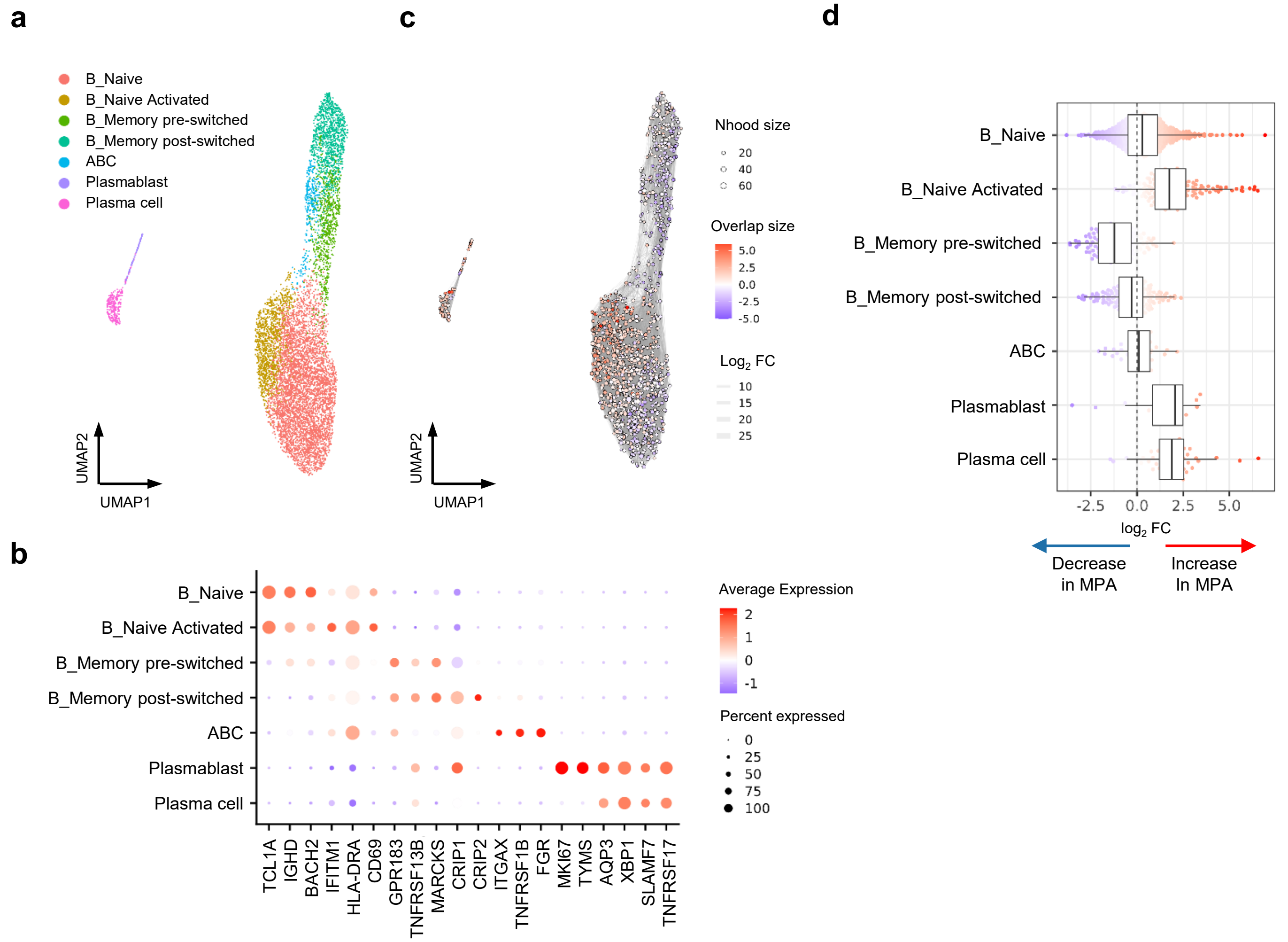

(a) UMAP plots showing the B cell subpopulations in a total of 15 study participants including healthy donors (n = 7) and patients with MPA (n = 8). Seven cellular clusters were identified; naïve B cells (B_Naïve), activated naïve B cells (B_Naïve Activated), pre-switched memory B cells (B_Memory pre-switched), post-switched memory B cells (B_Memory post-switched), age-associated B cells (ABC), plasmablasts (Plasmablast), and plasma cells (Plasma cell). **(b)** Balloon plot showing highly expressed gene in each B cell population shown in **(a)**. Balloon color indicates the averaged scaled expression of the indicated genes. Balloon size indicates the percentage of cells expressing the indicated genes. **(c)** Neighborhood graph of B cells using Milo differential abundance testing. Nodes represent neighborhoods from the B cell population. Colors indicate the log2-fold difference between patients with MPA and healthy donors. Neighborhoods that increased in patients with MPA are shown in red. Neighborhoods decreased in patients with MPA are shown in blue. **(d)** Beeswarm and box plots showing the distribution of $\log_2$-fold differences in neighborhoods in different cell type clusters. Colors are represented similarly to **(c).** Box plots are created in a similar fashion as in **Fig. 1f**.

Supplementary Fig. 6. Cydar analysis on monocyte subsets of CyTOF data.

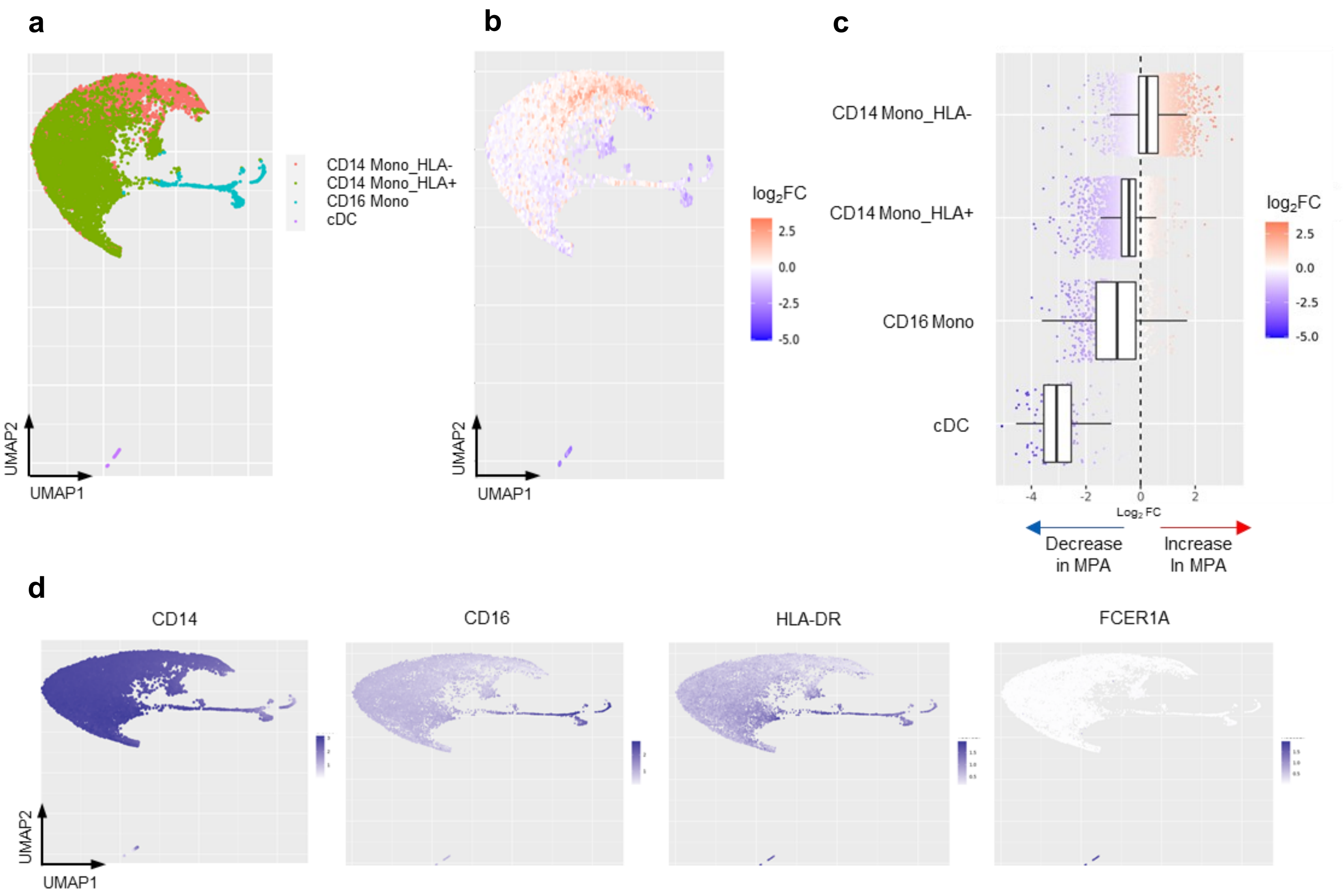

(a) UMAP plots showing CyTOF data of monocytes derived from a total of 15 study participants including healthy donors (n = 7) and patients with MPA (n = 8). Each cellular cluster was manually annotated based on surface marker expression. **(b)** Neighborhood graph of monocytes based on Cydar differential abundance testing. Nodes represent neighborhoods from the monocyte population. Colors indicate $\log_2$-fold differences between patients with MPA and healthy donors. Neighborhoods that increased in patients with MPA are shown in red. Neighborhoods decreased in patients with MPA are shown in blue. **(c)** Beeswarm and box plots showing the distribution of $\log_2$-fold differences in neighborhoods in different cell type clusters. Colors are represented similarly to **(b)**. **(d)** Feature plots of surface proteins based on Cydar analysis of monocytes. Each dot represents a hypersphere from the monocyte population. The average expression level of each protein on the cells included in each hypersphere was calculated. Box plots are created in a similar fashion as in **Fig. 1f**.

Supplementary Fig. 7. Cydar analysis on CD8+ T cell subsets of CyTOF data.

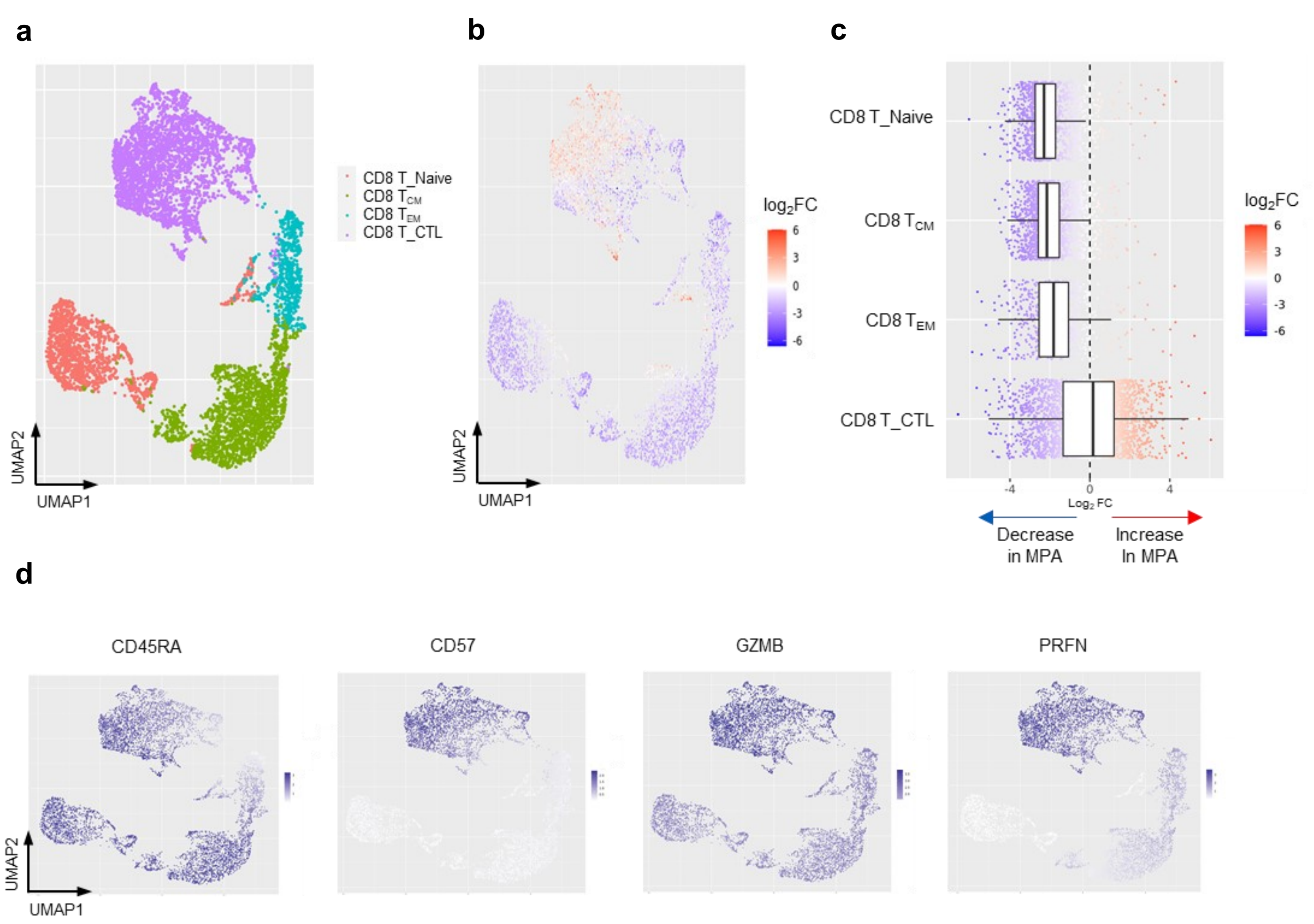

(a) UMAP plots showing CyTOF data of CD8^{+} T cells derived from a total of 15 study participants including healthy donors (n = 7) and patients with MPA (n = 8). Each cellular cluster was manually annotated based on surface marker expression. **(b)** Neighborhood graph of CD8^{+} T cells based on Cydar differential abundance testing. Nodes represent neighborhoods from the CD8^{+} T cell population. Colors indicate $\log_2$-fold differences between patients with MPA and healthy donors. Neighborhoods that increased in patients with MPA are shown in red. Neighborhoods decreased in patients with MPA are shown in blue. **(c)** Beeswarm and box plots showing the distribution of $\log_2$-fold differences in neighborhoods in different cell type clusters. Colors are represented similarly to **(b)**. **(d)** Feature plots of surface proteins based on Cydar analysis of CD8^{+} T cells. Each dot represents a hypersphere from the CD8^{+} T cell population. The average expression level of each protein on the cells included in each hypersphere was calculated. CD45RA and CD57 are surface proteins. GZMB and PRF1 are intracellular cytokines. GZMB: Granzyme B, PRF1: Perforin1. Box plots are created in a similar fashion as in **Fig. 1f**.

Supplementary Fig. 8. Heatmaps of interferon signature genes in each subset of PBMC.

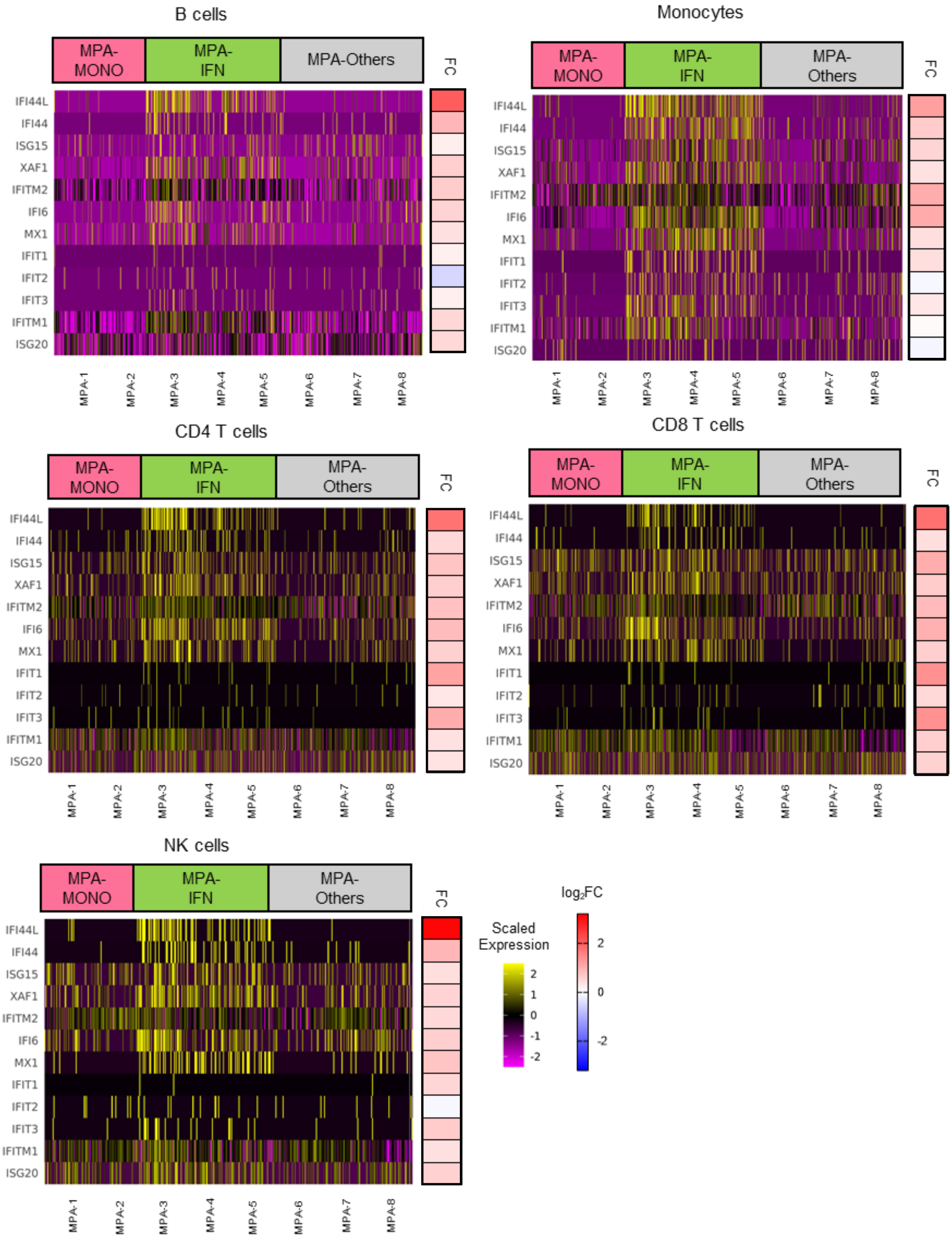

Heatmap showing the scaled expression of interferon signature genes (ISGs) in B cells, monocytes, CD4+ T cells, CD8+ T cells, and natural killer (NK) cells at the single-cell level. Each column represents a patient with MPA. Log_2-fold difference in the average expression of each ISG between MPA-MONO (MPA-1 and MPA-2) and MPA-ISG (MPA-3, MPA-4, and MPA-5) is shown.

Supplementary Fig. 9. Population and pathway analysis of B cells derived from each study participant.

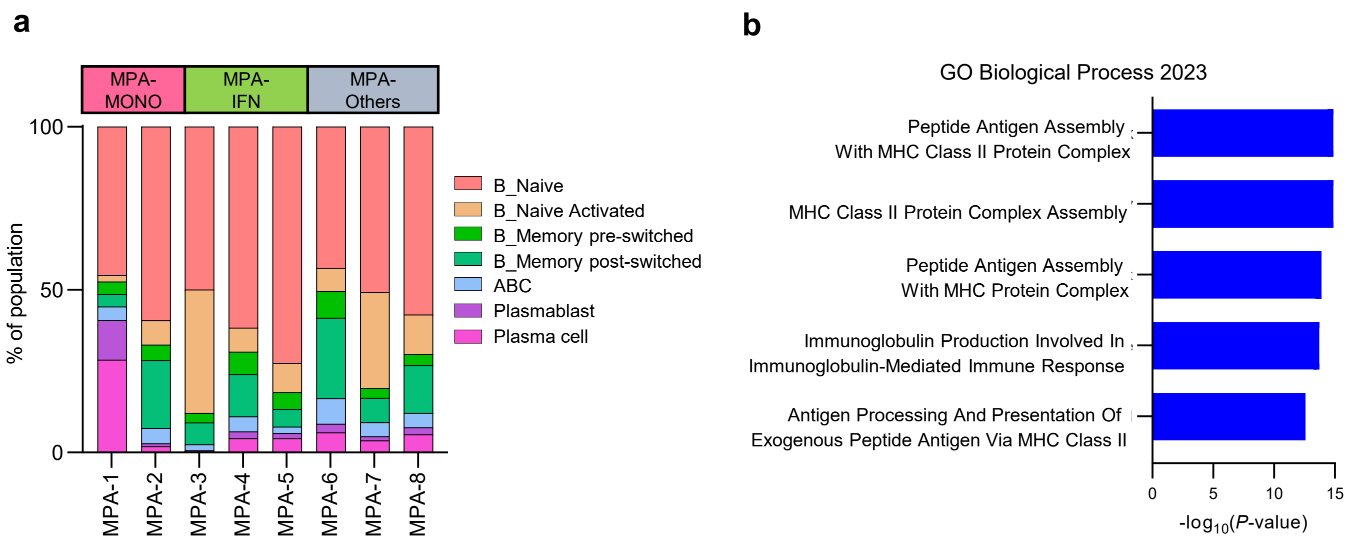

(a) Bar plots showing the proportion of each B cell subpopulation relative to total number of B cells. Each subpopulation was annotated in a similar fashion as in **Supplementary Fig. 5a**. **(b)** Gene set enrichment analysis of differentially expressed genes from patients in MPA-IFN group using GO Biological Process 2023. The pathway analysis was performed based on the top 50 genes significantly upregulated in B cells derived from patients in MPA-IFN group compared to patients in MPA-MONO. *P*-values for each pathway were calculated by Benjamini-Hochberg method.

Supplementary Fig. 10. Module scoring analysis of the monocyte subpopulations.

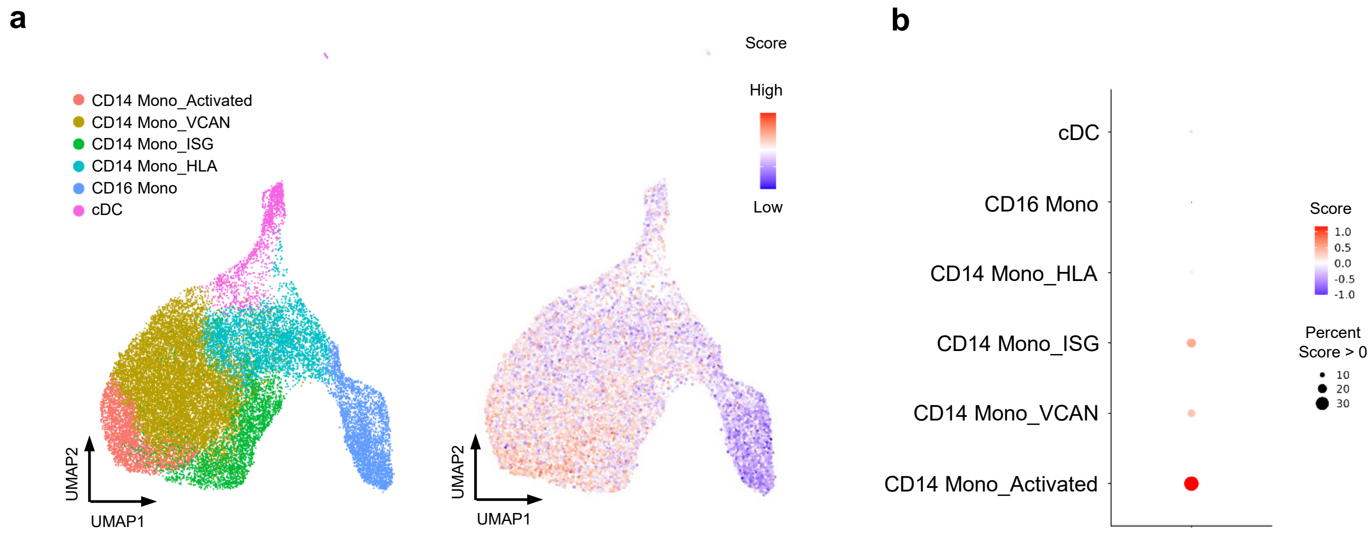

(a) Module scoring analysis of the monocyte subpopulations. The module score was calculated using gene signatures of a specific monocyte subset, previously reported as immature and newly mobilized from the bone marrow during inflammation (Reference No. 24). Each cell was colored according to the scaled module score. **(b)** Balloon plot showing the averaged module score in each monocyte subpopulation, as shown in **(a)**.

Supplementary Fig. 11. Correlation between clinical parameters and CITE-seq-based gene signature scores.

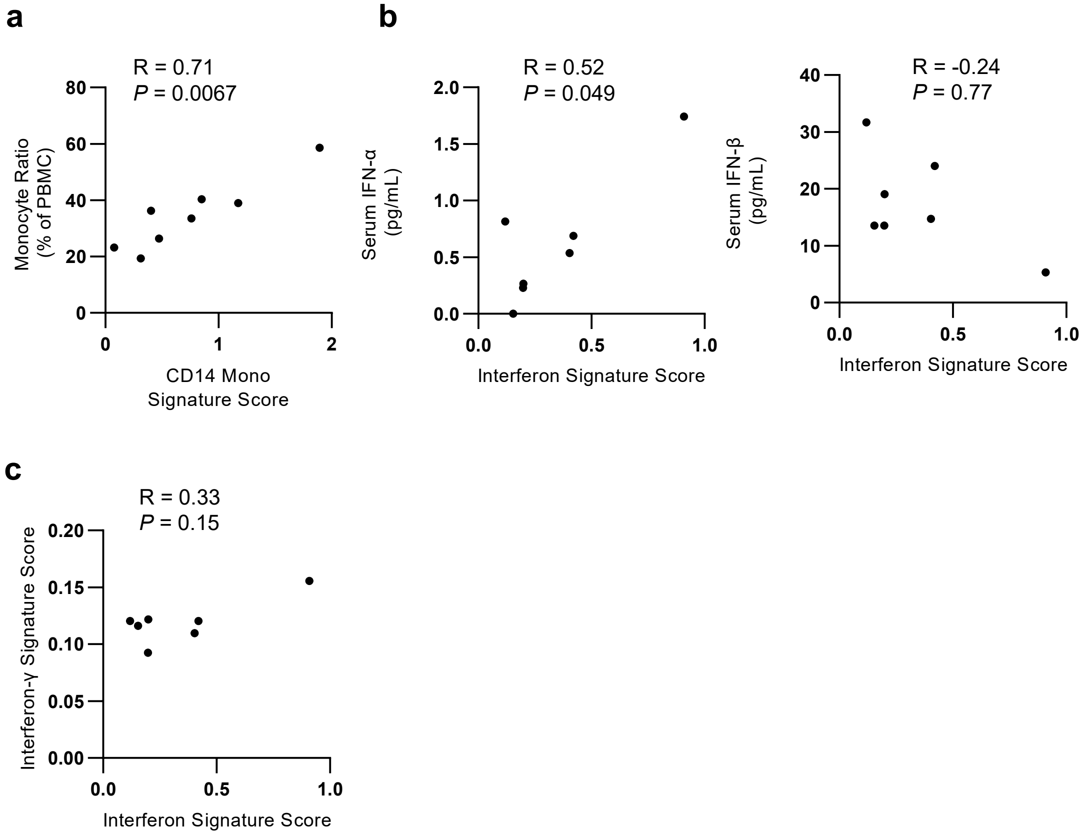

(a) Correlation between monocyte ratio in the blood cell count relative to the number of PBMCs and CD14^{+} monocyte signature scores. **(b)** Correlations between serum IFN-α / IFN-β concentrations and interferon signature gene scores. **(c)** Correlations between IFN-γ signature gene score and interferon signature gene scores. Correlations and *P*-values were quantified using Kendall's correlation coefficients (R).

Supplementary Fig. 12. Classification of the MPA-MONO and MPA-IFN groups among 28 patients with newly-onset MPA.

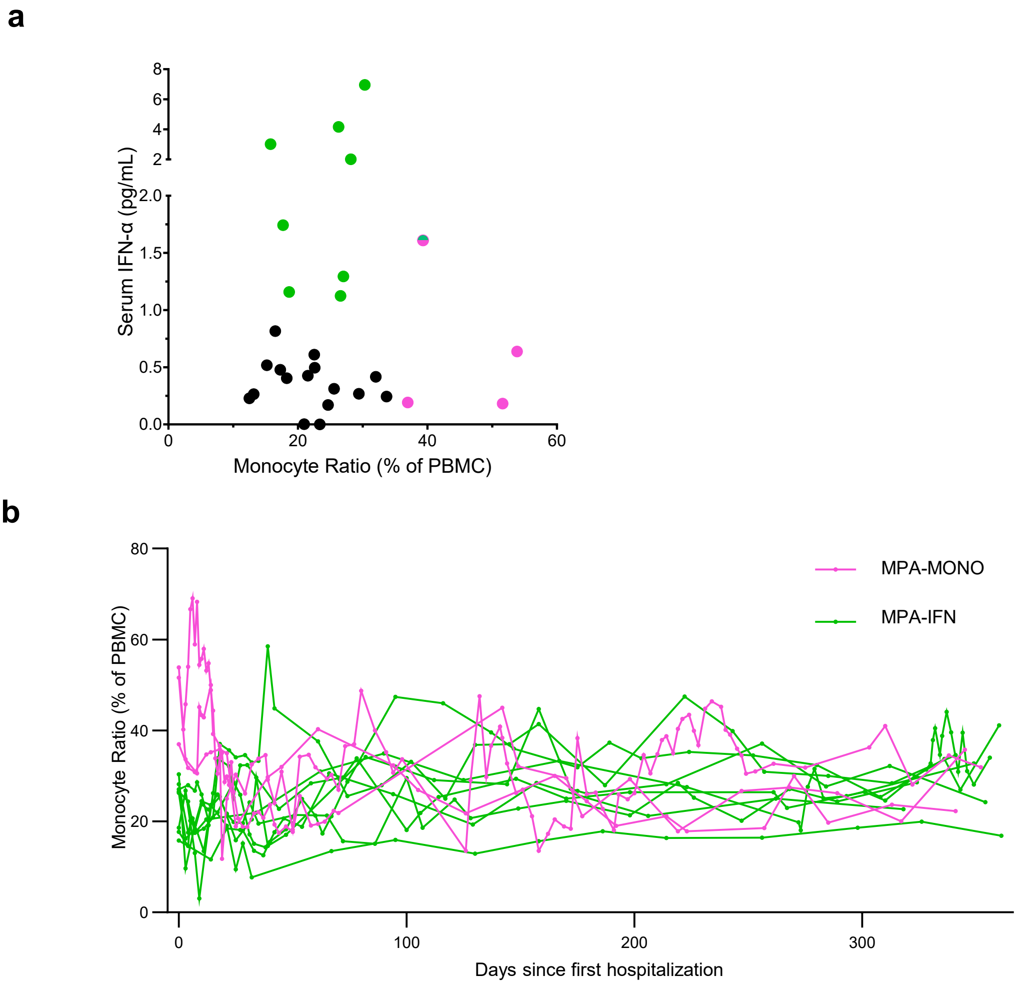

(a) Serum IFN-α concentrations and the percentage of monocytes among PBMCs in the complete blood count of samples from patients with MPA (only newly-onset patients, n = 28). The 3 samples with the highest percentage of monocytes were classified as MPA-MONO (pink colored dots). The 8 samples with the highest IFN-α concentrations were classified as MPA-IFN (green colored dots). **(b)** The monocyte ratio in newly-diagnosed MPA-MONO (n = 3) and MPA-IFN (n = 8) patients, tracked over a one-year period since first hospitalization.

Supplementary Fig. 13. Pre-processing of FCS files to select for live single cells.

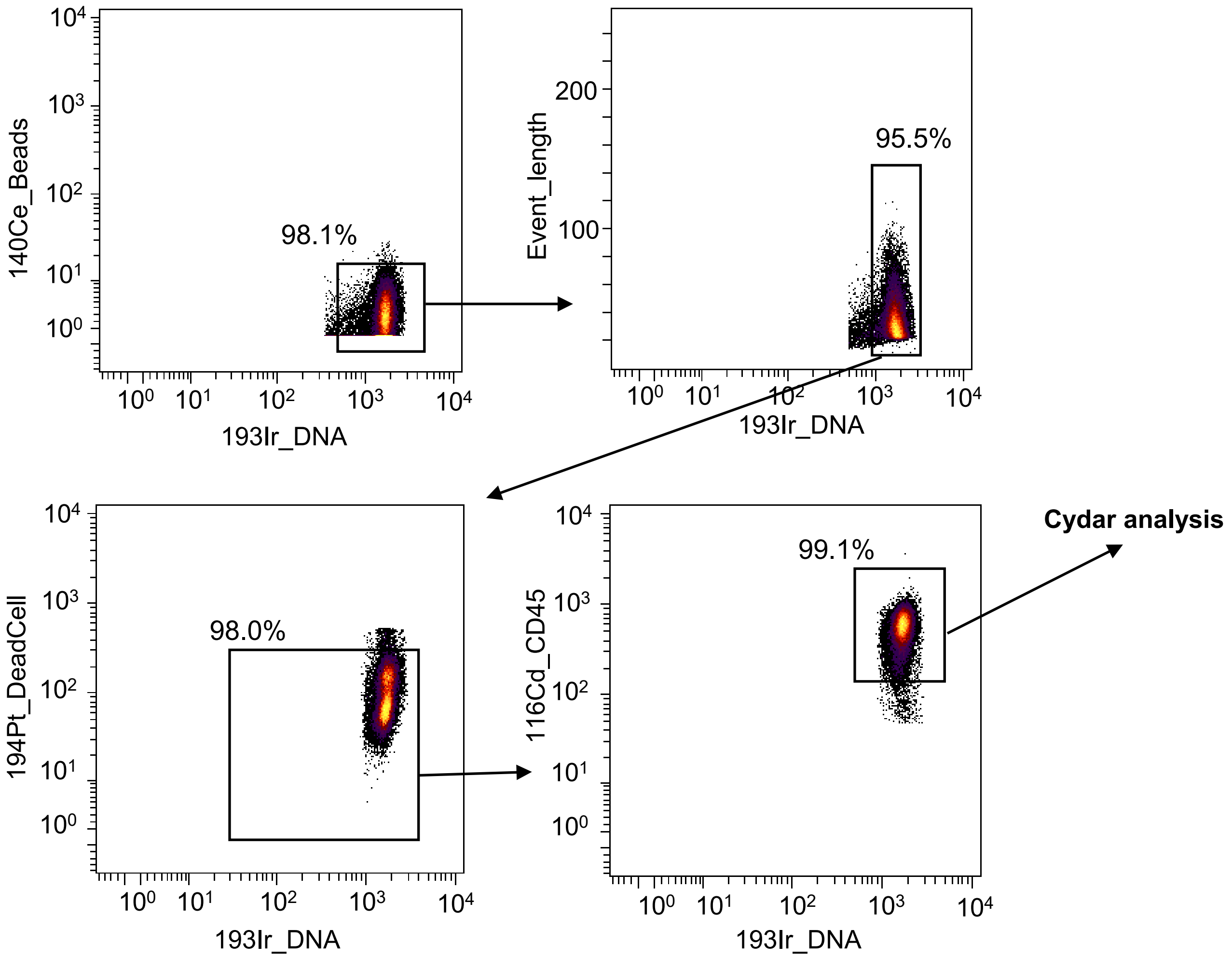

The gating strategy for pre-processing FCS files to remove normalization beads and debris, and to select for live single cells. FCS files containing only live cells were exported for subsequent Cydar analysis.
