# Supplementary material for: Single-cell multi-omics analysis identifies two distinct phenotypes of newly-onset microscopic polyangiitis
Source: Nat Commun. 2023 Oct 11;14:5789. doi: 10.1038/s41467-023-41328-0 (PMC10567716; doi:10.1038/s41467-023-41328-0)
Supplement: Supplementary file 3 — Reporting Summary [file 41467_2023_41328_MOESM3_ESM.pdf]

nature portfolio

Corresponding author(s): Masayuki Nishide

Last updated by author(s): Jul 6, 2023

Reporting Summary

Nature Portfolio wishes to improve the reproducibility of the work that we publish. This form provides structure for consistency and transparency in reporting. For further information on Nature Portfolio policies, see our Editorial Policies and the Editorial Policy Checklist.

Statistics

For all statistical analyses, confirm that the following items are present in the figure legend, table legend, main text, or Methods section.

n/a	Confirmed	
☐	☒	The exact sample size (*n*) for each experimental group/condition, given as a discrete number and unit of measurement
☐	☒	A statement on whether measurements were taken from distinct samples or whether the same sample was measured repeatedly
☐	☒	The statistical test(s) used AND whether they are one- or two-sided<br>*Only common tests should be described solely by name; describe more complex techniques in the Methods section.*
☐	☒	A description of all covariates tested
☐	☒	A description of any assumptions or corrections, such as tests of normality and adjustment for multiple comparisons
☐	☒	A full description of the statistical parameters including central tendency (e.g. means) or other basic estimates (e.g. regression coefficient) AND variation (e.g. standard deviation) or associated estimates of uncertainty (e.g. confidence intervals)
☐	☒	For null hypothesis testing, the test statistic (e.g. *F*, *t*, *r*) with confidence intervals, effect sizes, degrees of freedom and *P* value noted<br>*Give P values as exact values whenever suitable.*
☒	☐	For Bayesian analysis, information on the choice of priors and Markov chain Monte Carlo settings
☒	☐	For hierarchical and complex designs, identification of the appropriate level for tests and full reporting of outcomes
☒	☐	Estimates of effect sizes (e.g. Cohen's *d*, Pearson's *r*), indicating how they were calculated

Our web collection on statistics for biologists contains articles on many of the points above.

Software and code

Policy information about availability of computer code

Data collection	CyTOF software (v7.0.8493), CellRanger (v6.0.6), SoftMax Pro (v7.1)
Data analysis	Seurat (v4.2.0), miloR (v3.15), cytoNorm (v0.0.7), Enrichr (v3.1), cydar (v1.22.0), cytobank (v10.0), pROC (v1.18.0) and ggplot2 (v3.4.0) were R packages installed and used for analysis and visualization of CITE-seq and CyTOF data. All functions were derived from these packages. Graphpad Prism (v8) were used for data visualization.<br>The code is available at https://github.com/KeiNishim/MPA_scRNAseq

For manuscripts utilizing custom algorithms or software that are central to the research but not yet described in published literature, software must be made available to editors and reviewers. We strongly encourage code deposition in a community repository (e.g. GitHub). See the Nature Portfolio guidelines for submitting code & software for further information.

Data

Policy information about availability of data

All manuscripts must include a data availability statement. This statement should provide the following information, where applicable:

- Accession codes, unique identifiers, or web links for publicly available datasets
- A description of any restrictions on data availability
- For clinical datasets or third party data, please ensure that the statement adheres to our policy

We deposit the expression matrix data of CITE-seq of all the subject in this study, which is available at Genomic Expression Archive (GEA). Accession code is noted in

the 'Data availability' section.
The reference for cell type annotation of PBMC in scRNA-seq was obtained from satija lab (https://satijalab.org/seurat/articles/multimodal_reference_mapping.html.)
The GRCh38 reference genome was obtained from NCBI (https://www.ncbi.nlm.nih.gov/assembly/GCF_000001405.26/)

Human research participants

Policy information about studies involving human research participants and Sex and Gender in Research.

Reporting on sex and gender	In this study, sex of participants was determined based on self-report. No sex- and gender-based analyses have been performed because this study pursues the phenotype of the entire vasculitis regardless sex or gender.
Population characteristics	Eight patients with MPA (four females; median age, 72 years) and seven healthy donors (four females; median age, 62 years) were recruited for CITE-seq experiments. 43 patients with MPA (24 females; median age, 75 years) were additionally recruited to evaluate clinical and laboratory parameters.
Recruitment	Patients who visited Osaka university hospital were recruited after informed consent was provided in accordance with the Declaration of Helsinki and with approval from the ethics review board of the Graduate School of Medicine, Osaka University, Japan (No. 855).<br>There is no self-selection bias as all patients newly diagnosed as MPA and hospitalized in Osaka University Hospital from July, 2020 to. March, 2022 were recruited for the study.
Ethics oversight	Ethics review board of the Graduate School of Medicine, Osaka University, Japan (No. 855).

Note that full information on the approval of the study protocol must also be provided in the manuscript.

Field-specific reporting

Please select the one below that is the best fit for your research. If you are not sure, read the appropriate sections before making your selection.

☒ Life sciences ☐ Behavioural & social sciences ☐ Ecological, evolutionary & environmental sciences

For a reference copy of the document with all sections, see nature.com/documents/nr-reporting-summary-flat.pdf

Life sciences study design

All studies must disclose on these points even when the disclosure is negative.

Sample size	The sample size was not predetermined and all patients newly diagnosed as MPA and hospitalized in Osaka University Hospital from July, 2020 to. March, 2022 are recruited. We recruited eight patients with MPA and seven healthy donors.<br>Consequently, 109,350 cells for CITE-seq and 737,794 cells for CyTOF were used for data analysis. Sample size were sufficient to adequately detect compositional change of rare population by clustering and different abundance analysis
Data exclusions	No data were excluded.
Replication	All findings were based on statistical analysis of a large patient cohort. There was no replication cohort.<br>All data including multi-omics analyses were processed twice for quality control and all attempts at replication were successfully.
Randomization	We did not apply randomization of the samples because no intervention was conducted in our study.
Blinding	We did not apply blinding of the samples because no intervention was conducted in our study.

Reporting for specific materials, systems and methods

We require information from authors about some types of materials, experimental systems and methods used in many studies. Here, indicate whether each material, system or method listed is relevant to your study. If you are not sure if a list item applies to your research, read the appropriate section before selecting a response.

Materials & experimental systems

n/a	Involved in the study
[ ]	[x] Antibodies
[x]	[ ] Eukaryotic cell lines
[x]	[ ] Palaeontology and archaeology
[x]	[ ] Animals and other organisms
[ ]	[x] Clinical data
[x]	[ ] Dual use research of concern

Methods

n/a	Involved in the study
[x]	[ ] ChIP-seq
[ ]	[x] Flow cytometry
[x]	[ ] MRI-based neuroimaging

Antibodies

Antibodies used

Please see our supplementary tables 9 and 11.

Validation

CITE-seq antibodies are validated by BioLegend, as stated in the website that they are tested by PCR and sequencing to confirm the oligonucleotide barcodes. They are also tested by flow cytometry to ensure the antibodies recognize the proper cell populations. CyTOF antibodies are validated by Fluidigm, as stated in the website that they are quality control tested by CyTOF analysis of stained cells using the appropoate positive and negative cell staining and/or activation controls.

Clinical data

Policy information about clinical studies

All manuscripts should comply with the ICMJEguidelines for publication of clinical research and a completedCONSORT checklist must be included with all submissions.

Clinical trial registration

N/A

Study protocol

N/A

Data collection

N/A

Outcomes

N/A

Flow Cytometry

Plots

Confirm that:

- [x] The axis labels state the marker and fluorochrome used (e.g. CD4-FITC).
- [x] The axis scales are clearly visible. Include numbers along axes only for bottom left plot of group (a 'group' is an analysis of identical markers).
- [x] All plots are contour plots with outliers or pseudocolor plots.
- [x] A numerical value for number of cells or percentage (with statistics) is provided.

Methodology

Sample preparation

For human PBMCs collection, whole blood (20 mL) was collected into a Na-heparin blood collection tube (Terumo, VP-H070K). PBMCs were separated using Leucosep (Greiner, 22788-013). PBMCs were washed and resuspended with Cellbanker 1plus (ZENOAQ, CB023) to a concentration of 1.0 × 107 cell/mL before being stored at −150℃.

PBMCs were thawed and prepared to a concentration of 1 × 107 cell/mL. Next, they were cultured in RPMI-1640 medium for 6 hours at 37℃ with GolgiStop supplementation (BD bioscience, 554724). To limit the batch effect, we barcoded each sample based on combinations of seven types of anti-CD45 antibodies derived from MCP9 Labeling Kit (Standard Biotools, 201111A) 30 minutes before the endpoint of the culture. Cell-ID Cisplatin (Fluidigm, 201064) (2 μM) was added 15 minutes before the endpoint of the culture. All barcoded samples were then combined and stained with antibodies specific for surface markers for 30 minutes at room temperature. To normalize the data across multiple batches, we combined control PBMCs (Cellular Tchnology Limited, CTL-UP1) across all batches. The samples were fixed with 1 mL of Maxper Fix and Perm buffer (Fluidigm, 201067) for 30 minutes at 4℃. Cells were stained in 1 mL of Foxp3 Fixation/Permeabilization buffer (eBioscience, 00-5523-00) with antibodies specific for intracellular cytokines and Cell-ID intercalator-Ir (Fluidigm, 201192A) for 30 minutes at room temperature. The antibodies used for CyTOF are shown in Supplementary Table 11. The antibodies were derived from Human Maxpar Direct Immune Profiling Assay (Standard Biotools, 201334), Human PB Phenotyping Panel Kit (Standard Biotools, 201304), Human T-Cell Phenotyping Panel Kit (Standard Biotools, 201305), and Human Intracellular Cytokine I Panel Kit (Standard Biotools, 201308) and used at the concentration specified in the kits. The samples were suspended in a total of 10% Four Element Calibration Beads (Fluidigm, 201078) with Cell Acquisition Solution (Fluidigm, 201244).

Instrument

Helios CyTOF system (Fluidigm, 107002)

Software

CyTOF software (Fluidigm, ver 7.0.8493)

Cell population abundance	No sorted cell fraction underwent experiments.
Gating strategy	Cells are selected using 193Ir_DNA positive and 140Ce_Beads negative. Live cell are selected by removing 194Pt_DeadCell strong populations. Then samples are selected using Cd_CD45 barcode positive cells. Selected cells are used for further analysis using R cydar algorithms. Gating strategy is provided in Extended Data Fig.13.

[x] Tick this box to confirm that a figure exemplifying the gating strategy is provided in the Supplementary Information.
